# Differences in prevalence and risk factors of non-communicable diseases between young people living with HIV (YLWH) and young general population in Cambodia

**DOI:** 10.1371/journal.pone.0269989

**Published:** 2022-06-21

**Authors:** Kennarey Seang, Marjan Javanbakht, Sung-Jae Lee, Ron Brookmeyer, Phearavin Pheng, Phalla Chea, Vonthanak Saphonn, Pamina M. Gorbach

**Affiliations:** 1 Grant Management Office, University of Health Sciences, Phnom Penh, Cambodia; 2 Department of Epidemiology, University of California Los Angeles, Los Angeles, California, United States of America; 3 Department of Biostatistics, University of California Los Angeles, Los Angeles, California, United States of America; 4 International Relation Division, University of Health Sciences, Phnom Penh, Cambodia; 5 Rectorate, University of Health Sciences, Phnom Penh, Cambodia; University of New Mexico Health Sciences Center, UNITED STATES

## Abstract

Understanding non-communicable diseases (NCDs) among young people living with HIV (YLWH) is critical given the potential for aging-associated comorbidities resulting from HIV, especially in Cambodia where such data are limited. Therefore, we examined the prevalence and correlates of NCDs in YLWH and compared it to a nationally representative sample of young people not otherwise infected. We collected data from a sample of 370 YLWH aged 18–29 years attending three HIV clinics in Cambodia between 2019 and 2020. Our comparison group were 486 young people who participated in the Ministry of Health/WHO 2016 Noncommunicable Disease Risk Factor Surveillance (STEP survey). Both surveys used a standardized questionnaire to collect information on lifestyle factors and World Health Organization protocols for physical and biochemical measurements. We compared the prevalence of diabetes, hypertension, and high cholesterolemia between the two groups and examined the relationship between these conditions and HIV. We found 16 (4%), 22 (6%), and 72 (20%) had diabetes, hypertension, and high cholesterolemia, respectively, among YLWH, compared to 4 (1%), 22 (4%), and 49 (11%) among the general population. In logistic regression, YLWH were at higher odds of diabetes/prediabetes and high cholesterolemia compared with the young general population, aOR = 6.64 (95% CI 3.62–12.19) and aOR = 7.95 (95% CI 3.98–15.87), respectively. Our findings demonstrate that YLWH in Cambodia face multiple metabolic disorders and NCDs despite their young age and that accessible screening measures and treatment for these conditions are needed in order to combat NCDs in the future.

## Introduction

Cambodia is home to roughly 16 million people, with approximately 65,000 people estimated to be living with HIV [1]. Since early 2000, the HIV infection rate in Cambodia continues to decrease [2] and the estimated HIV prevalence is less than 1% as of 2016 [3]. Young people living with Human Immunodeficiency Virus or HIV (YLWH) in Cambodia are, therefore, mostly those who had been perinatally infected and under antiretroviral therapy (ART) for a long period of time. Based on 2016 estimates, the ART coverage among PLWH is 80% [3]. Starting in 2000, the country underwent a demographic and epidemiological transition, with mortality from non-communicable diseases or NCDs such as cardiovascular disease and diabetes, overtaking those from infectious diseases [4]. Despite this, the transition of public health research focus from infections to NCDs has been slow, resulting in very few research studies addressing NCDs over the years [4].

The increase in ART coverage is marked by an increase in life expectancy among people living with HIV (PLWH) and, to various extent, in other age-related comorbidities and NCDs [5–10]. Increasing evidence, mostly from studies conducted in the West, implicates HIV infection and ART in the increased risk of multiple chronic metabolic disorders including the glucose [11] and lipid disorders for older PLWH [12, 13]. The infection, the virus and the treatments might act independently and/or by influencing known traditional risk factors for NCDs [14, 15]. As a result, NCD risk might be higher among PLWH and this had been reported in a number of studies conducted in high-income countries (HICs) [16–19]. However, country-specific epidemiological data are still lacking in many low-middle-income countries (LMICs) [20–22]. Differences in the distribution of traditional and HIV-specific risk factors for NCDs across country settings also suggest that findings from one LMIC may not be applicable to other regions/LMICs [19–23], marking the need for country-specific data.

Because the risk profile and prevalence of NCDs among YLWH had not been comprehensively assessed in Cambodia, we aimed to examine the differences in the distribution of NCDs, as well as NCD risk factors, among YLWH and the young general population. We focus on adolescents for multiple reasons. First, the period during adolescence and young adulthood is considered one of the critical periods for targeting behavioural or medical interventions, as these could maximize the late adulthood health benefits, i.e. preventing the development of chronic diseases [24]. Second, many of NCD-related behaviours as well as their risk factors are quite prevalent among this population group; according to the World Health Organization (WHO), “over 150 million young people smoke, 81% adolescents don’t get enough physical activity, and 11.7% of adolescents partake in heavy episodic drinking” [25]. Lastly, although the overall HIV prevalence is low, it is high among certain subgroups of young population [3], all of whom will be aging and living with HIV their whole life, so addressing NCDs among young population today helps steer the future course of NCD morbidity and mortality.

## Materials and methods

### Study setting and design

We conducted a cross sectional study of 370 YLWH receiving care at three HIV clinics in Phnom Penh (Cambodia). Participants of both sexes were considered eligible if they were between the ages of 18 and 29 years, were HIV-positive, and had a medical check-up visit during the study period (November 2019-February 2020). The three clinics which served as the site for this study are designated “OI/ART” sites for Opportunistic Infections and Antiretroviral Therapy and serve an estimated 20,000 patient population. These clinics offer both HIV testing and treatment services and receive patients mainly from Phnom Penh (capital city), although there are patients from the provinces as well. These clinics are only three among over 10 clinics in Phnom Penh (or three out of about 70 across Cambodia) which offer HIV services.

Data for the general population comparison group used as part of this study were obtained from those collected as part of the STEPwise Approach to Noncommunicable Disease Risk Factor Surveillance or STEP survey in 2016. The STEP survey was first launched in 2010 by the Cambodia’s Ministry of Health (MOH), in collaboration with the WHO as well as other local and international partnering institutions as part of the attempt to describe the burden of NCDs and their risk factors among the general Cambodian population [26, 27]. Details of the STEP survey are described elsewhere, but briefly the 2016 STEPS was a multi-stage cluster sampling survey, with the selection of villages across Cambodia using probability-proportional-to-size (PPS) in the first stage, followed by the simple random sampling (SRS) of households and then participants in the next two stages, using standardized questionnaire and measurement protocols. Those eligible for inclusion in this analysis were from the STEP household survey and included both males and females between the age of 18–29 from all provinces in Cambodia in 2016 (n = 486).

### Data collection tools and process

We conducted a Computer-Assisted-Person-Interview (CAPI) using tablets for the 370 YLWH recruited. We used a comparable survey tool as the STEPs survey and included additional questions on HIV-specific factors, such as ART regimen and duration of ART use. Questions on physical activity, alcohol drinking and smoking had been adapted to include local activities, such as farming and household chores, local alcoholic drinks and tobacco products, respectively.

Prior to survey administration, we collected biometric information including blood pressure as well as blood glucose and total cholesterol. We employed digital scales to measure weight, OMRON^®^ Upper Arm Blood Pressure Monitor, measuring tapes and BeneCheck^®^ rapid testing devices. Of note, blood pressure was measured three times, five minutes apart, and the average of the three was then calculated. The rapid blood tests (for glucose and total cholesterol levels) took less than a minute and participants fasting status was recorded. After these measurements, we provided snacks and drinks to each participant and the results of their measurements were also given to them. In the end, the data collector would conduct a face-to-face interview with each individual using a structured questionnaire which was administered via tablet.

### Ethics approval

The current study was approved by the Institutional Review Board of the University of California Los Angeles (UCLA IRB#19–000272) and the National Ethics Committee for Health Research in Cambodia (NECHR #129) in 2019. No identifying information were collected and all study participants provided verbal and written consent according to the approved study protocols.

### Analytic methods

We calculated mean, median, and frequency distributions for behavioural and clinical factors and differences between YLWH and the general population group was evaluated using t-test, chi-square tests, as appropriate. The primary outcomes of interest included diabetes/pre-diabetes, high and borderline cholesterolemia, and hypertension. Diabetes was defined as a fasting glucose ≥ 126 mg/dL or post-prandial glucose ≥ 200mg/dL and/or on anti-diabetic medications. Prediabetes was defined as a fasting glucose between 101–125 mg/dL or post-prandial glucose between 140–199 mg/dL. Those who had a mean systolic blood pressure (SBP) ≥ 140 mmHg and/or mean diastolic blood pressure (DBP) ≥ 90 mmHg and/or on anti-hypertensive drugs were defined as hypertensive, while prehypertension included participants with a mean SBP between 120–139 mmHg and/or mean DBP between 80–89 mmHg. High cholesterolemia was based on fasting (n = 659) and non-fasting blood (n = 135) samples and was defined as a total cholesterol ≥ 240 mg/dL and/or on cholesterol lowering drugs with those with total cholesterol between 200–239 mg/dL being defined as having borderline hypercholesterolemia. About 7% (n = 62) of the study volunteers had not been tested for glycemia and cholesterolemia (fear of finger pricks and being late for work were among the most common reasons).

Independent variables of interest included demographic characteristics as well as smoking and drinking status, physical activity and dietary habits. Smokers were defined as those who were current users of tobacco products. Heavy drinkers were defined as those who during the past week had four standard drinks or more on any one occasion or 14 standard drinks or more in total (men), or three standard drinks or more on any one occasion or seven standard drinks or more in total (women). Fruit and vegetable consumption was defined as any one-day consumption on a typical week within the past year. Physical activity (recreation- or work-related) included locally-adapted activities, such as farming and household chores. Low physical activity included those who reported less than 75 minutes of work or recreation activity of vigorous intensity per week or less than 150 minutes of recreation activity of moderate intensity per week. Body Mass Index (BMI) classification was based on Asian-specific cut-offs–underweight refers to BMI < 18.5, normal weight refers to BMI between 18.5 and 23, overweight and obese refer to BMI ≥ 23 and BMI ≥ 25, respectively. The definitions and classification used were based on multiple sources [28–32].

We fitted a separate logistic regression model to examine the association between each of these conditions and HIV; we reported both the crude and adjusted odds ratios (ORs), the latter controlled for age, sex, residence location (Phnom Penh vs. provinces), behavioural risk factors (smoking, heavy drinking, less than five servings of fruits and vegetables, low physical activity) and BMI. These factors are considered confounders based on prior knowledge, literature review and findings from univariate analyses. All analyses used STATA version 14 (StataCorp, College Station, TX).

## Results

### Socio-demographics characteristics of the study sample

Table 1 describes the socio-demographic and NCD risk factor characteristics of the study population. We found that 254 (70%) of participants who are living with HIV were male, whereas, female participants accounted for 344 (70%) of the general population (p < 0.001). The average age was 23 years (SD 3.2) among YLWH and 24 years (SD 3.3) among the general population (p < 0.001). In terms of education, more YLWH reported having had completed at least high school, 210 (58%), while only 72 (15%) of the young general population reported so. We also found a difference in terms of area of residence (location) between the two groups with 468 (95%) of those in the general population group reporting living in the provinces, compared to only 58 (23%) of YLWH (p < 0.001).

**Table 1:** Key characteristics of study participants, STEPS, Cambodia, 2016 and 2019/2020.

	YLWH (*n* = 370)		Young General Population (*n* = 486)		p
	*n*	*%*	*n*	*%*	
Socio-demographics					
Gender					
Male	254	70.8	146	29.8	< 0.001
Female	105	29.2	344	70.2	
Age					
Mean, SD	(23.1, 3.2)		(24.1, 3.3)		< 0.001
18–24	226	62.8	242	49.8	< 0.001
25–29	134	37.2	244	50.2	
Education					
None or less than primary	7	1.9	177	36.1	< 0.001
Completed primary	41	11.4	136	27.8	< 0.001
Completed secondary	101	28.1	105	21.4	0.002
Completed high school or higher	210	58.5	72	14.7	< 0.001
Occupation					
Salary employed	211	58.8	64	13.1	< 0.001
Self-employed	70	19.5	312	63.7	< 0.001
Unemployed	78	21.7	114	23.3	0.60
Marital status					
Single	315	87.7	149	30.4	< 0.001
Married+cohabiting	40	11.1	322	65.7	< 0.001
Other	4	1.1	19	3.4	0.01
Residence types					
Province	85	23.5	468	95.5	< 0.001
Phnom Penh	277	76.5	22	4.5	
**NCD behavioural risk factors**					
Smokers	17	4.7	32	6.5	0.26
Heavy drinkers	52	19.6	37	23.3	0.37
< Five servings of fruits and vegetables	232	65.9	245	50.1	< 0.001
Low physical activity	81	38.9	44	20.4	< 0.001
**NCDs**					
Body Mass Index (BMI)					
Normal weight	210	58.2	251	55.6	0.006
Underweight	94	26.0	91	20.2	
Overweight and obese	57	15.8	109	24.2	
Blood glucose					
Prediabetes^a^	120	34.0	23	5.2	< 0.001
Diabetes^a^	16	4.5	4	0.9	0.001
Fasting blood glucose^b^ (mean, SD)	(105.6, 26.3)		(80.8, 19.6)		< 0.001
Blood pressure					
Prehypertension	114	31.6	111	23.0	0.008
Hypertension	22	6.1	22	4.5	0.32
SBP^b^ (mean, SD)	(113.4, 12.0)		(110.0, 11.3)		< 0.001
DBP^b^ (mean, SD)	(75.2, 8.1)		(72.4, 9.1)		< 0.001
Total cholesterol					
Borderline-high^a^	34	12.2	82	21.0	0.003
Borderline-high^c^	22	12.9	82	21.0	0.02
High^a^	72	20.4	49	11.2	< 0.001
High^c^	47	21.7	49	11.2	< 0.001
Fasting total cholesterol^b^ (mean, SD)	(189.4, 63.1)		(179.7, 49.4)		0.08

SD, Standard Deviation.

^a^Included ALL participants.

^b^Excluded those currently on treatment for each specified condition.

^c^Included ONLY fasting participants.

### NCD risk factor distribution

The prevalence of smoking was less than 10% in both groups (p = 0.26) and heavy alcohol consumption differed only slightly among the two groups, 20% (n = 52) among YLWH vs. 23% (n = 37) among the general population (p = 0.37) (Table 1). Although low fruit and vegetable consumption and low physical activity were more common among YLWH compared to the general population, the number of young people who were overweight and obese was higher in the young general population group, 24% (n = 109), compared to the group living with HIV16% (n = 57) (p < 0.01).

While a smaller proportion of YLWH were overweight or obese, compared to young people from the general population, the prevalence of NCDs and certain factors predisposing this group to NCDs were higher among those who were HIV-positive (Table 1). The prevalence of diabetes and hypertension was 4% (n = 16) and 6% (n = 22), respectively, among YLWH, compared to 1% (n = 4) and 4% (n = 22), among the general population. Additionally, a substantially higher proportion of YLWH had pre-diabetes, elevated blood pressure (i.e., pre-hypertension), and high cholesterol when compared to young people from the general population.

**Fig 1:**
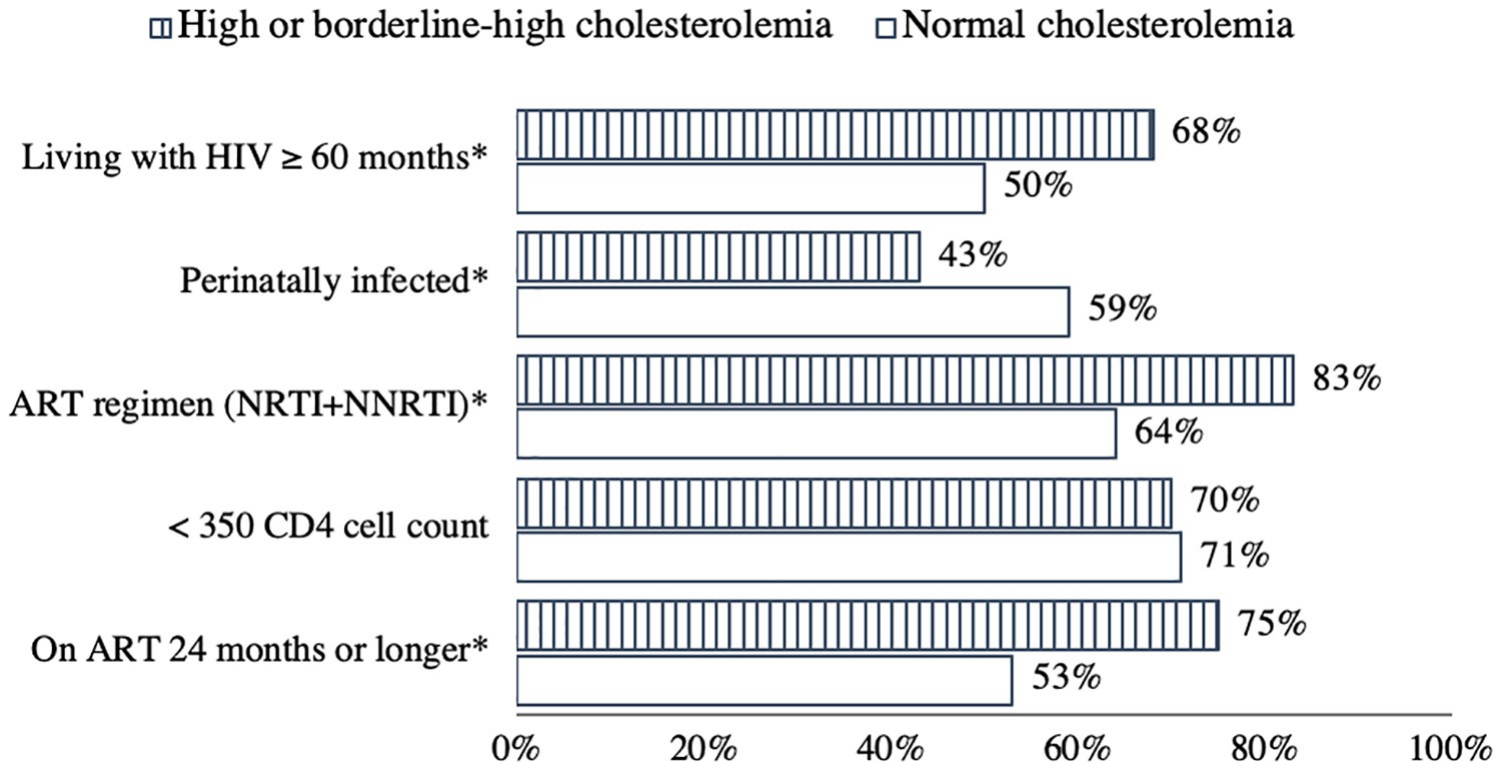
Distribution of cholesterolemia status by HIV-specific factors. * *P<* 0.05 (Chi-square test of association) Source: Data from the 2016 UHS (n = 486) and 2019/2020 (n = 370) STEP surveys, Cambodia.

A subanalysis of HIV-infected individuals focusing on HIV-specific factors revealed that high or borderline-high cholesterolemia was associated with longer duration of ART use (24 months or longer), combination Nucleoside Reverse Transcriptase Inhibitors (NRTI) and Non-nucleoside Reverse Transcriptase Inhibitors (NNRTI) therapy, perinatal HIV infection and longer duration of HIV infection (60 months or longer) (Fig 1). For instance, we found that the prevalence of high or borderline-high cholesterolemia was 75% among those on ART for more than 24 months compared to only 25% among those on ART for less than 24 months (p ≤ 0.001). Other than cholesterolemia, no other conditions had been found to be marginally associated with any of the HIV-specific parameters. It should be noted that the combination therapy (NNRTI and NRTI) was the most common regimen among patients in Cambodia and PI-based regimen is relatively less common.

### Factors associated with NCDs

#### Outcome: Diabetes or prediabetes

We grouped diabetes or prediabetes in order to ensure sufficient number of outcomes across exposure status and other adjusting variables (the number of diabetic cases alone is low in both groups). In multivariable analyses we found that young people who were HIV-infected had an increased odd of diabetes/prediabetes. In particular, we found YLWH had a nearly 7-fold increased odds of diabetes/prediabetes when compared to young people from the general population even after adjusting for other factors including sex, age, current residence location, behavioural risk factors and BMI, aOR = 6.64 (95% CI 3.62–12.19) (Table 2).

**Table 2:** Association between diabetes/prediabetes and HIV, STEPS, Cambodia, 2016 and 2019/2020.

	Diabetes or Prediabetes	
	Crude OR (95% CI)	Adjusted^a^ OR (95% CI)
HIV+ (ref. young general population)	9.59 (6.15–14.95)	6.64 (3.62–12.19)
Female (ref. male)	0.29 (0.20–0.42)	0.67 (0.44–1.03)
Age	0.94 (0.90–1.00)	0.96 (0.91–1.03)
Current address in Phnom Penh (ref. in provinces)	5.44 (3.76–7.89)	1.33 (0.81–2.21)
2 or more behavioural risk factors^b^	1.78 (1.17–2.71)	1.17 (0.73–1.86)
BMI (ref. normal weight)		
Underweight	0.62 (0.40–0.99)	0.49 (0.29–0.81)
Overweight and obese	0.92 (0.59–1.44)	1.19 (0.72–1.98)

CI, Confidence interval.

Diabetes: fasting glucose ≥ 126 mg/dL or post-prandial glucose ≥ 200mg/dL and/or on anti-diabetic drugs.

Prediabetes: fasting glucose between 101–125 mg/dL or post-prandial glucose between 140–199 mg/dL.

^a^Adjusted for sex, age, residence location, behavioural risk factors and BMI.

^b^Behavioural risk factors include smoking, heavy alcohol consumption, less than five servings of fruit and vegetable consumption and low physical activity.

**Fig 2:**
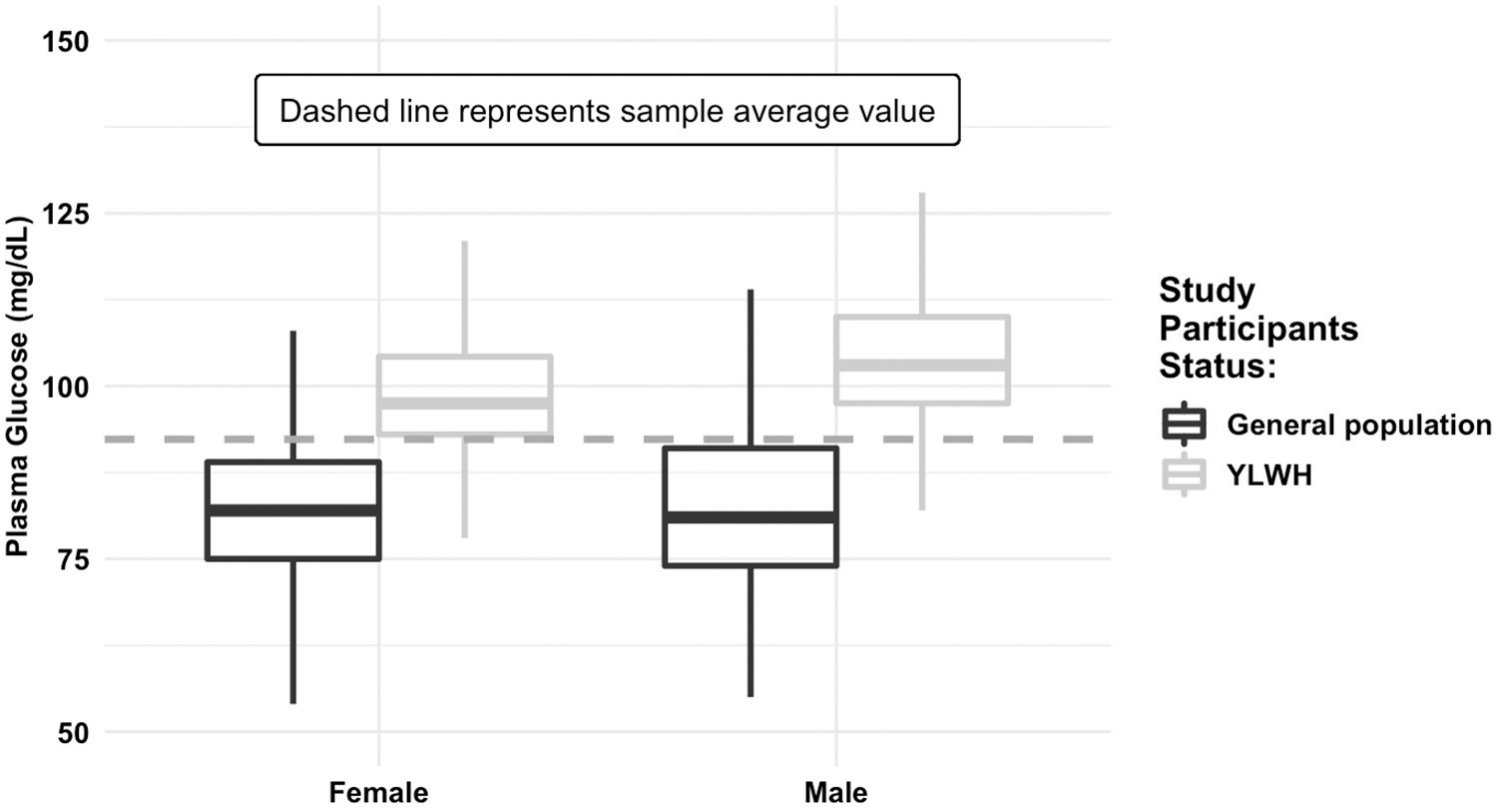
Plasma glucose levels among (fasting) study participants, stratified by BMI. Source: 2016 UHS (n = 486) and 2019/2020 (n = 370) STEP surveys, Cambodia.

**Fig 3:**
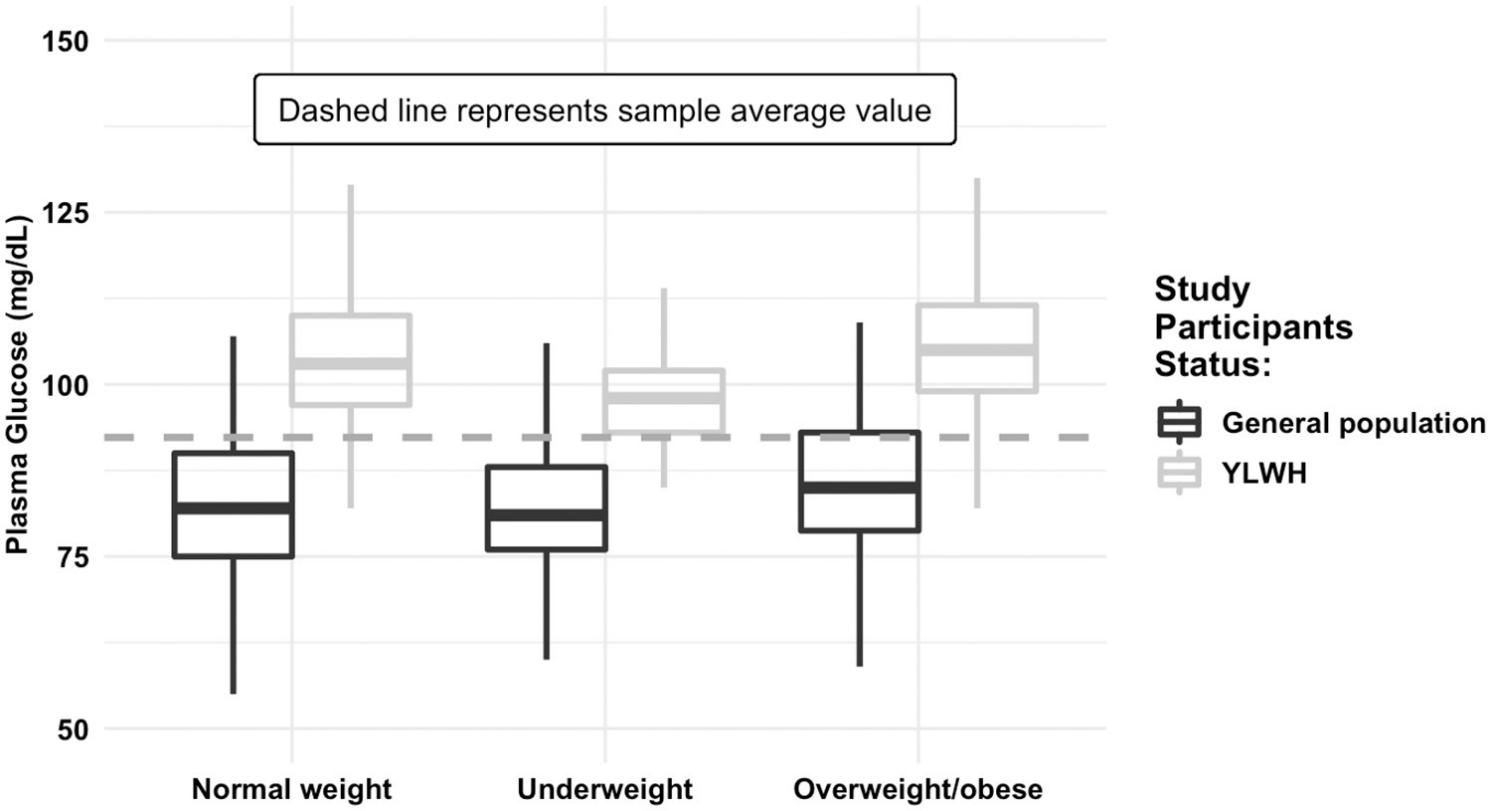
Plasma glucose levels among (fasting) study participants, stratified by sex. Source: 2016 UHS (n = 486) and 2019/2020 (n = 370) STEP surveys, Cambodia.

Boxplots depicting the relationship between fasting plasma glucose levels and HIV, are presented in Figs 2 and 3. Regardless of BMI and sex, YLWH appeared to have a higher fasting plasma glucose level compared to the young general population.

#### Outcome: Hypertension and prehypertension

In crude analysis, the crude ORs appeared to suggest a positive association between both hypertension and prehypertension and HIV, cOR = 1.60 (95% CI 1.17–2.18) and cOR = 1.36 (95% CI 0.74–2.50), respectively. However, after adjustment was made (Table 3), YLWH were no longer at higher odds of these conditions compared with the young general population, aOR = 1.01 (95% CI 0.59–1.71) for prehypertension and aOR = 0.41 (95% CI 0.13–1.27) for hypertension.

**Table 3:** Association between hypertension and prehypertension and HIV, STEPS, Cambodia, 2016 and 2019/2020.

	Hypertension		Prehypertension	
	Crude OR (95% CI)	Adjusted^a^ OR (95% CI)	Crude OR (95% CI)	Adjusted^a^ OR (95% CI)
HIV+ (ref. young general population)	1.36 (0.74–2.50)	0.41 (0.13–1.27)	1.60 (1.17–2.18)	1.01 (0.59–1.71)
Female (ref. male)	0.36 (0.18–0.70)	0.38 (0.17–0.83)	0.31 (0.22–0.43)	0.28 (0.19–0.42)
Age	1.12 (1.02–1.23)	1.09 (0.99–1.21)	1.06 (1.01–1.12)	1.06 (1.01–1.12)
Current address in Phnom Penh (ref. in provinces)	1.91 (1.04–3.51)	3.54 (1.21–10.41)	1.41 (1.02–1.94)	1.18 (0.70–1.97)
2 or more behavioural risk factors^b^	2.12 (1.08–4.17)	1.70 (0.82–3.56)	1.19 (0.79–1.78)	0.89 (0.57–1.40)
BMI (ref. normal weight)				
Underweight	0.66 (0.21–2.01)	0.73 (0.24–2.28)	0.54 (0.35–0.84)	0.62 (0.39–0.97)
Overweight and obese	5.27 (2.70–10.28)	5.18 (2.58–10.38)	1.94 (1.31–2.86)	2.15 (1.41–3.28)

CI, Confidence interval.

Hypertension: mean SBP ≥ 140 mmHg and/or mean DBP ≥ 90 mmHg and/or on anti-hypertensive drugs.

Prehypertension: mean SBP between 120–139 mmHg and/or mean DBP between 80–89 mmHg.

^**a**^Adjusted for sex, age, residence location, behavioural risk factors and BMI.

^**b**^Behavioural risk factors include smoking, heavy alcohol consumption, less than five servings of fruit and vegetable consumption and low physical activity.

#### Outcome: High and borderline-high cholesterol

Among young people, HIV status was independently associated with elevated cholesterol levels after adjusting for sociodemographic and other risk characteristics. YLWH almost eight times (after adjustment) more likely than the young general population participants to have high cholesterolemia, aOR = 7.95 (95% CI 3.98–15.87) (Table 4). The odds of borderline-high cholesterolemia did not appear to be different among the two groups, aOR = 0.98 (95% CI 0.45–2.15). As noted in the methods section, cholesterol testing was inclusive of those who fasted as well as those who did not fast prior to testing. In subanalyses, the associations between total cholesterol and HIV remained largely unaffected with or without inclusion of those who did not fast before rapid blood tests (results not shown).

**Table 4:** Association between high and borderline-high cholesterolemia and HIV, STEPS, Cambodia, 2016 and 2019/2020.

	High cholesterolemia		Borderline-high cholesterolemia	
	Crude OR (95% CI)	Adjusted^a^ OR (95% CI)	Crude OR (95% CI)	Adjusted^a^ OR (95% CI)
HIV+ (ref. young general population)	2.04 (1.37–3.02)	7.95 (3.98–15.87)	0.52 (0.34–0.81)	0.98 (0.45–2.15)
Female (ref. male)	2.52 (1.65–3.83)	5.10 (3.02–8.60)	3.43 (2.20–5.35)	3.59 (2.09–6.14)
Age	1.01 (0.95–1.07)	1.05 (0.97–1.12)	1.11 (1.04–1.19)	1.09 (1.01–1.16)
Current address in Phnom Penh (ref. in provinces)	1.53 (1.03–2.26)	0.78 (0.43–1.42)	0.64 (0.41–1.01)	1.29 (0.61–2.71)
2 or more behavioural risk factors^b^	1.12 (0.68–1.86)	1.38 (0.78–2.45)	0.42 (0.21–0.84)	0.72 (0.35–1.49)
BMI (ref. normal weight)				
Underweight	0.88 (0.52–1.49)	0.69 (0.39–1.21)	0.36 (0.19–0.68)	0.32 (0.16–0.62)
Overweight and obese	1.10 (0.65–1.87)	1.32 (0.75–2.33)	1.24 (0.75–2.20)	1.03 (0.61–1.72)

CI, Confidence interval.

High cholesterolemia: total cholesterol between ≥ 240 g/dL and/or on cholesterol lowering drugs.

Borderline-high cholesterolemia: total cholesterol between 200–239 g/dL.

^a^Adjusted for sex, age, residence location, behavioural risk factors and BMI.

^b^Behavioural risk factors include smoking, heavy alcohol consumption, less than five servings of fruit and vegetable consumption and low physical activity.

## Discussion

Our findings demonstrate that young people living with HIV in Cambodia, mainly in the capital city, face multiple metabolic disorders and NCDs despite their young age and lower prevalence of traditional risk factors such as smoking and alcohol consumption compared with general population of the same age group. The prevalence of glucose disorders (diabetes and prediabetes) and high cholesterolemia were significantly higher among our YLWH compared with the young general population. Additionally, our results suggest that HIV infection and ART might have important roles in contributing to the early development of chronic diseases, such as diabetes, prediabetes and high cholesterolemia, in addition to the conventional NCD risk factors.

The prevalence of diabetes found in our study (4.6%) was comparable to that reported in the study conducted in Lubowa which reported a prevalence of 4.7% [33]. However, the study in Lubowa defined diabetes with a much higher threshold of fasting plasma glucose (140 mg/dL or higher), resulting in a small number of diabetes cases [33]. Furthermore, the Lubowa study was inclusive of a much older study population with only 11% of participants being under the age of 30 year. Given that adults between the ages of 45 and 65 are the most common age group for new diabetes diagnoses, at least in the US [34], our findings highlight the potential magnitude of the problem as this population ages.

We noted a relatively high prevalence of high cholesterolemia in our sample of YLWH both when compared to participants from the general population as well as other studies conducted among those living with HIV [5]. This could be partially explained by an increase in Protease-Inhibitor-based regimen (PI) use among children (from only 5% in 2009 to 13% in 2016, based on reports from the National Center for HIV/AIDS, Dermatology and STDs (NCHADS) [3, 35]. Given that PIs have been linked with dyslipidaemia, regions such as Cambodia, with a high prevalence of perinatally transmitted HIV-infection will have to give special consideration, both from a clinical and public health perspective [36, 37].

The distribution of other factors, such as urban vs rural residence location and BMI might also be contributing to some extent to the overall findings of the present study. We note that with the exception of hypertension and prehypertension, the association between BMI and NCDs appeared to be rather weak or null. The lack of obvious association between overweight/obese BMI with high blood sugar and cholesterol when HIV status was also accounted for in the regression model is more likely due to relatively smaller number of overweight/obese YLWH compared to the young general population in the same BMI category. This was rather contradictory, because despite having higher prevalence of overweight/obese BMI, the majority of our young general population lived in the provinces, where the exposure to fast food chains and other unhealthy foods should have been less common (especially in Cambodia). However, this might explain a higher prevalence of diabetes and prediabetes and high cholesterolemia among YLWH compared to the young general population. Regardless, these secondary associations should be interpreted with care, the relationship structure we assumed when running these models depicted the selected NCDs and HIV status as the outcome and exposure of interest, interpreting the associations between covariates and outcome(s) implied a different relationship structure.

The study should be interpreted with consideration to a number of limitations. First, by design, we understand that we have to be careful when interpreting the association between HIV and NCDs in causal terminology. Nevertheless, these results were consistent with that of multiple studies including those of longitudinal nature, such as the Multi-AIDS Cohort Study (MACS) [38]. In addition, about half of YPLW in the study sample were perinatally-infected and the average duration of HIV infection was about 9 years. Taking this as well as participant’s age into consideration, we can reasonably assume that metabolic disorders measured as part of this study were likely to have occurred after the onset of HIV infection and ART. We also saw that the NCD prevalence among those perinatally infected and behaviourally infected did not differ, particularly in the case of diabetes/prediabetes and hypertension/prehypertension. Second, the general population was assumed to be HIV-negative, and given the low HIV prevalence among the general population in Cambodia (< 1%), the amount of misclassification (if any) should be negligible. Lastly, there were several covariates related to the NCDs that were not measured in the original STEP survey conducted among the general population, including oral contraceptive use (for women) and birth history (prematurity or low birth weight). As a result, we could not account for any of these factors in the analysis. Nevertheless, the focus of the STEP survey is the modifiable lifestyle factors, many of which were indeed included in logistic regression model. Moreover, the majority of our YLWH were males and although we didn’t show in results, over 80% of our female participants reported no use of oral contraceptives at the time of data collection.

## Conclusions

While the impact of HIV-infection and its association with NCDs have been previously noted, our findings confirm this in a much younger population and add to the limited data on NCDs and HIV in low- and middle-income countries. Understanding the contributions of these HIV-specific factors to an increase in chronic disease risk suggests putting in place early proper screening as well as treatment measures for certain metabolic disorders (mainly prediabetes, diabetes and high total cholesterolemia) targeted young people living with HIV may be an effective strategy to combat NCD morbidity and mortality in Cambodia. Further investigations including detailed lipid profile assessment (including LDL Cholesterol, HDL Cholesterol and triglycerides) might be necessary to fully comprehend the extent to which HIV-specific factors affect various forms of fats. Furthermore, the Cambodian ART regimen guidelines need further consideration; studies designed specifically to assess the effects of various ART drugs on these metabolic chronic disorders are needed, especially that of glucose and cholesterol.

## Supporting information

S1 File (DOCX)

S2 File (DOCX)

## References

[1] Central Intelligence Agency (CIA). The World Factbook: Cambodia. The World Factbook: Central Intelligence Agency (USA). 2018.

[2] Population Reference Bureau (PRB). Spread of HIV is slowing in Cambodia 2003 [updated 2003; cited 2021 March]. https://www.prb.org/spreadofhivisslowingincambodia/.

[3] Ministry of Health and the National Center for HIV/AIDS, Dermatology and STDs. Annual Report 2016. National Center for HIV/AIDS, Dermatology and STDs. 2017.

[4] Goyet S, Touch S, Ir P, SamAn S, Fassier T, Frutos R, et al. Gaps between research and public health priorities in low income countries: evidence from a systematic literature review focused on Cambodia. Implement Sci. 2015;10:32. doi: 10.1186/s13012-015-0217-1 . 25889672 PMC4357145

[5] Chhoun P, Ngin C, Tuot S, Pal K, Steel M, Dionisio J, et al. Non-communicable diseases and related risk behaviors among men and women living with HIV in Cambodia: findings from a cross-sectional study. Int J Equity Health. 2017;16(1):125. doi: 10.1186/s12939-017-0622-y . 28705242 PMC5513209

[6] Phillips A, Baker J, Lundgren J. Are antiretrovirals enough for people living with HIV? Lancet. 2013;382(9903):1466–7. doi: 10.1016/S0140-6736(13)62072-3 . 24182530

[7] Munderi P, Grosskurth H, Droti B, Ross DA. What are the essential components of HIV treatment and care services in low and middle-income countries: an overview by settings and levels of the health system? AIDS. 2012;26 Suppl 2:S97–S103. doi: 10.1097/QAD.0b013e32835bdde6 . 23303438

[8] Chhoun P, Tuot S, Harries AD, Kyaw NTT, Pal K, Mun P, et al. High prevalence of non-communicable diseases and associated risk factors amongst adults living with HIV in Cambodia. PloS one. 2017;12(11):e0187591. doi: 10.1371/journal.pone.0187591 . 29121661 PMC5679628

[9] Mathabire Rucker SC, Tayea A, Bitilinyu-Bangoh J, Bermudez-Aza EH, Salumu L, Quiles IA, et al. High rates of hypertension, diabetes, elevated low-density lipoprotein cholesterol, and cardiovascular disease risk factors in HIV-infected patients in Malawi. AIDS. 2018;32(2):253–60. doi: 10.1097/QAD.0000000000001700 . 29135581 PMC5757671

[10] Feinstein MJ H P, Benjamin LA, Bloomfield GS, Currier JS, Freiberg MS, Grinspoon SK, et al. AHA Statement on HIV and Cardiovascular Disease: Clinical Strategies and Future Challenges. Circulation. 2019. 10.1161/CIR.0000000000000695 PMC7993364 31154814

[11] Pebody R. Type 2 diabetes and HIV. NAM-aidsmap. 2017.

[12] Muller EV, Gimeno SGA. Risk factors for cardiovascular disease in HIV/AIDS patients treated with highly active antiretroviral therapy (HAART) in the central-southern region of the state of Parana—Brazil. Cien Saude Colet. 2019;24(5):1903–14. Epub 2019/06/06. doi: 10.1590/1413-81232018245.16682017 . 31166523

[13] Suligoi B, Virdone S, Taborelli M, Frova L, Grande E, Grippo F, et al. Excess mortality related to circulatory system diseases and diabetes mellitus among Italian AIDS patients vs. non-AIDS population: a population-based cohort study using the multiple causes-of-death approach. BMC Infect Dis. 2018;18(1):428. Epub 2018/08/30. doi: 10.1186/s12879-018-3336-x . 30153797 PMC6114052

[14] Hemkens LG, Bucher HC. HIV infection and cardiovascular disease. Eur Heart J. 2014;35(21):1373–81. doi: 10.1093/eurheartj/eht528 . 24408888

[15] Ho JE, Hsue PY. Cardiovascular manifestations of HIV infection. Heart. 2009;95(14):1193–202. Epub 2009/07/01. doi: 10.1136/hrt.2008.161463 . 19564432

[16] Smit M, Brinkman K, Geerlings S, Smit C, Thyagarajan K, Sighem A, et al. Future challenges for clinical care of an ageing population infected with HIV: a modelling study. Lancet Infect Dis. 2015;15(7):810–8. doi: 10.1016/S1473-3099(15)00056-0 . 26070969 PMC4528076

[17] Castilho JL, Shepherd BE, Koethe J, Turner M, Bebawy S, Logan J, et al. CD4+/CD8+ ratio, age, and risk of serious noncommunicable diseases in HIV-infected adults on antiretroviral therapy. AIDS. 2016;30(6):899–908. doi: 10.1097/QAD.0000000000001005 . 26959354 PMC4785819

[18] De Francesco D, Wit FW, Cole JH, Kootstra NA, Winston A, Sabin CA, et al. The ’COmorBidity in Relation to AIDS’ (COBRA) cohort: Design, methods and participant characteristics. PloS one. 2018;13(3):e0191791. doi: 10.1371/journal.pone.0191791 . 29596425 PMC5875743

[19] Raghavan A, Rimmelin DE, Fitch KV, Zanni MV. Sex Differences in Select Non-communicable HIV-Associated Comorbidities: Exploring the Role of Systemic Immune Activation/Inflammation. Curr HIV/AIDS Rep. 2017;14(6):220–8. doi: 10.1007/s11904-017-0366-8 . 29080122 PMC6007989

[20] Petersen M, Yiannoutsos CT, Justice A, Egger M. Observational research on NCDs in HIV-positive populations: conceptual and methodological considerations. J Acquir Immune Defic Syndr. 2014;67 Suppl 1:S8–16. doi: 10.1097/QAI.0000000000000253 . 25117964 PMC4317266

[21] Deeks SG, Lewin SR, Havlir DV. The end of AIDS: HIV infection as a chronic disease. Lancet. 2013;382(9903):1525–33. doi: 10.1016/S0140-6736(13)61809-7 . 24152939 PMC4058441

[22] Narayan KM, Miotti PG, Anand NP, Kline LM, Harmston C, Gulakowski R 3rd, et al. HIV and noncommunicable disease comorbidities in the era of antiretroviral therapy: a vital agenda for research in low- and middle-income country settings. J Acquir Immune Defic Syndr. 2014;67 Suppl 1:S2–7. doi: 10.1097/QAI.0000000000000267 . 25117958

[23] Matanje Mwagomba BL, Ameh S, Bongomin P, Juma PA, MacKenzie RK, Kyobutungi C, et al. Opportunities and challenges for evidence-informed HIV-noncommunicable disease integrated care policies and programs: lessons from Malawi, South Africa, Swaziland and Kenya. AIDS. 2018;32 Suppl 1:S21–S32. doi: 10.1097/QAD.0000000000001885 . 29952787

[24] Baird J, Barker M, Fall CHD; Hanson M, Harvey NC, Inskip HM, Kumaran K, et al. Developmental Origins of Health and Disease: A Lifecourse Approach to the Prevention of Non-Communicable Diseases. Healthcare. 2017;5(1). doi: 10.3390/healthcare5010014 28282852 PMC5371920

[25] Baker R, Taylor E, Essafi S, Jarvisd JD & Odokd C. Engaging young people in the prevention of noncommunicable diseases [cited 2020 April]. https://www.who.int/bulletin/volumes/94/7/16-179382.pdf. 10.2471/BLT.16.179382 PMC4933150 27429484

[26] World Health Organization. STEPwise Approach to Noncommunicable Disease Risk Factor Surveillance (STEPS) [cited 2019 January]. https://www.who.int/teams/noncommunicable-diseases/surveillance/systems-tools/steps. 10.2105/AJPH.2015.302962 PMC4695948 26696288

[27] Prevalence of Non-communiucable Disease Risk Factors in Cambodia. Cambodia Ministry of Health; University of Health Sciences; World Health Organization. 2010.

[28] Centers for Disease Control and Prevention. Adult Tobacco Use Information. 2017 [cited 2018 October 5]. https://www.cdc.gov/nchs/nhis/tobacco/tobacco_glossary.htm.

[29] Mayo Clinic. High Cholesterol. 2019 [updated February 23 2019; cited 2019 June 12]. https://www.mayoclinic.org/diseases-conditions/high-blood-cholesterol/diagnosis-treatment/drc-20350806.

[30] National Institute on Alcohol Abuse and Alcoholism (NIAAA). [cited 2018 October 5]. https://www.niaaa.nih.gov/alcohol-health/overview-alcohol-consumption/moderate-binge-drinking.

[31] World Health Organization. Raised blood pressure [cited 2019 June 13]. https://www.who.int/gho/ncd/risk_factors/blood_pressure_prevalence_text/en/.

[32] World Health Organization. Global Recommendations on Physical Activity for Health: 18–64 years old. 2011 [cited 2019 March 13]. https://www.who.int/dietphysicalactivity/physical-activity-recommendations-18-64years.pdf?ua=1.

[33] Kansiime S, Mwesigire D, Mugerwa H. Prevalence of non-communicable diseases among HIV positive patients on antiretroviral therapy at joint clinical research centre, Lubowa, Uganda. PloS one. 2019;14(8):e0221022. Epub 2019/08/10. doi: 10.1371/journal.pone.0221022 . 31398244 PMC6688817

[34] Centers for Disease Control and Prevention (CDC). National Diabetes Statistics Report 2020: Estimate of Diabetes and Its Burden in the United States. 2020 [cited 2021 February]. https://www.cdc.gov/diabetes/pdfs/data/statistics/national-diabetes-statistics-report.pdf.

[35] Ministry of Health/National Center for HIV/AIDS, Dermatology and STDs (MOH/NCHADS). Annaul Report 2009. 2009.

[36] Vaishnav J, Mody FM, Martin SS, Blumenthal RS. Cardiovascular Risk Assessment & Prevention in Patients With HIV: Should We Be More Aggressive With Statin Therapy in this Special Population? JACC. 2017.

[37] Feeney ER, Mallon PW. HIV and HAART-Associated Dyslipidemia. Open Cardiovasc Med J. 2011;5:49–63. doi: 10.2174/1874192401105010049 . 21643501 PMC3106351

[38] Palella FJ Jr., Phair JP. Cardiovascular disease in HIV infection. Curr Opin HIV AIDS. 2011;6(4):266–71. doi: 10.1097/COH.0b013e328347876c . 21546831 PMC3501268

