# Supplementary material for: Differences in prevalence and risk factors of non-communicable diseases between young people living with HIV (YLWH) and young general population in Cambodia
Source: PLoS One. 2022 Jun 21;17(6):e0269989. doi: 10.1371/journal.pone.0269989 (PMC9212152; doi:10.1371/journal.pone.0269989)
Supplement: S1 File — (DOCX) [file pone.0269989.s001.docx]

**Non-Communicable Diseases and Their Risk Factors**

**Among Young Cambodian Population**

For more information or inqueries, please contact the principle investigator:

**Kennarey Seang**, PhD Candidate in Epidemiology, UCLA Fielding School of Public Health, University of California Los Angeles, USA

**Mobile:** +855-99-714 779

**E-mail:** seang.kennarey@gmail.com|kennareyseang@ucla.edu

**SURVEY INFORMATION**

| Contacts and Location | |
| --- | --- |
| Respondent ID | └─┴─┴─┘ |
| VCCT/ART Site Name | Name of site………………………………….. |
| Date of Interview | DD MM YYYY  └─┴─┘└─┴─┘└─┴─┴─┘ |
| Interviewer Code | └─┴─┘ |
| Consent and Date | |
| Consent read and obtained | 1. Yes 2. No (End) |
| Time of interview (24h clock) | Hours : minutes └─┴─┘:└─┴─┘ |
| Checker  Date | Name……………………………………………  DD MM YYYY  └─┴─┘└─┴─┘└─┴─┴─┘ |

**PART I: STEPS 1-3**

**STEPS 1: Demographic Information**

| **Code** | **Questions** | **Response** |
| --- | --- | --- |
| Q 1 | Sex (record male or female as observed) | 1. Male 2. Female |
| Q 2 | Date of birth | MM YYYY  └─┴─┘└─┴─┴─┘ (Go to Q 4) |
| Q 3 | How old are you? | └─┴─┘years |
| Q 4 | Marital status | 1. Single 2. Married 3. Separated 4. Divorced 5. Widowed 6. Cohabiting   88. Refuse to answer |
| Q 5 | What is the highest level of education that you attained? | 1. No schooling 2. Completed Primary 3. Completed Secondary 4. Completed high school 5. Completed undergraduate degree 6. Graduate degree or higher   88. Refuse to answer |
| Q 6 | What is your main occupation? | 1. Government employee 2. Non-government employee 3. Self-employed 4. Student 5. Housewife 6. Retired 7. Unemployed 8. Other: specify: ………………………   88. Refuse to answer |
| Q 7 | How many people older than 18 years, including yourself, live in your household? | Number of people └─┴─┘ |
| Q 8 | Taking the past year, can you tell me what the average earnings of the household have been (in Riel)?  *(RECORD ONLY ONE, NOT ALL 3)* | Per week:…………………….. (Go to Q10)  Per month:………………….. (Go to Q10)  Per year:……………………… (Go to Q10) |
| Q 9 | If you don’t know the amount, can you give an estimate of the annual household income if I read some options to you? Is it  *(READ OPTIONS)* | 1. ≤ 1 050 000 Riels  2. More than 1 050 000   to 4 000 000 Riels   1. More than 4 000 000   to 17 500 000 Riels   1. More than 17 500 000   to 21 875 000 Riels   1. More than 21 875 000 Riels   77. Don’t Know  88. Refuse to answer |

**STEPS 1: Behavioral Measurements**

***1). Tobacco use***

Now I am going to ask you some questions about tobacco use.

| **Code** | **Questions** | **Response** |
| --- | --- | --- |
| Q 10 | Do you currently smoke or use any tobacco products such as cigarettes, cigars, pipes, snuff, and chew tobacco?  *(USE SHOWCARD)* | 1. Yes 2. No (Go to Q 14) |
| Q 11 | Do you currently smoke tobacco products daily? | 1. Yes 2. No |
| Q 12 | How old were you when you first started smoking or using tobacco? | └─┴─┘years  99. Don’t remember |
| Q 13 | On average, how many of the following do you smoke each day? | Daily Weekly  1. Manufactured  cigarettes └─┴─┴─┘..└─┴─┴─┘  2. Hand rolled  cigarettes └─┴─┴─┘..└─┴─┴─┘  3. Pipe fillings of  tobacco └─┴─┴─┘..└─┴─┴─┘  4. Cigars, cheroots,  cigarillos └─┴─┴─┘..└─┴─┴─┘  5. Others └─┴─┴─┘..└─┴─┴─┘  (Specify):………………………………………….. |
| Q 14 | If not a current smoker, did you ever smoke any tobacco products? | 1. Yes 2. No (Go to Q 16) |
| Q 15 | If yes, did you smoke daily? | 1. Yes 2. No   99. Don’t remember |
| Q 16 | Do you currently use any smokeless tobacco such as snuff or chewing tobacco? | 1. Yes 2. No (Go to Q 18) |
| Q 17 | If yes, on average how many times a day do you use…  (record for each type) | Daily Weekly  1. Snuff, by mouth └─┴─┴─┘..└─┴─┴─┘  2. Snuff, by nose └─┴─┴─┘..└─┴─┴─┘  3. Chewing tobacco└─┴─┴─┘..└─┴─┴─┘  4. Others └─┴─┴─┘..└─┴─┴─┘  (Specify):……………………………………………… |
| Q 18 | During the past 30 days, did someone smoke in your home? | 1. Yes 2. No |

***2). Alcohol Consumption***

The next questions ask about the consumption of alcohol.

| **Code** | **Questions** | **Response** |
| --- | --- | --- |
| Q 19 | Have you ever consumed a drink that contains alcohol such as beer, wine, spirit, fermented palm juice?  *(USE SHOWCARD OR SHOW EXAMPLES*) | 1. Yes 2. No (Go to Q 22) |
| Q 20 | Have you consumed alcohol within the past 12 months? | 1. Yes 2. No (Go to Q 22) |
| Q 21 | In the past 12 months, how frequently have you had at least one standard alcoholic drink?  *(USE SHOWCARD)* | 1. Daily 2. 5-6 days/week 3. 3-4 days/week 4. 1-2 days/week 5. 1-3 days/ month 6. Less than once a month |
| Q 22 | Have you consumed any alcohol within the past 30 days? | 1. Yes 2. No (Go to Q 27) |
| Q 23 | During the past 30 days, on how many occasions did you have at least one standard alcoholic drink? | Number └─┴─┘  77. Don’t know |
| Q 24 | During the past 30 days, when you drank alcohol, how many standard drinks on average did you have during one drinking occasion?  *(USE SHOWCARD)* | Number └─┴─┘  77. Don’t know |
| Q 25 | During the past 30 days, what was the largest number of standard drinks you had on a single occasion, counting all types of alcoholic drinks together? | Largest number └─┴─┘  77. Don’t know |
| Q 26 | During the past 30 days, how many times did you have  six or more standard drinks in a single drinking occasion? | Number of times └─┴─┘  77. Don’t know |
| Q 27 | During each day of the past 7 days, how many standard drinks of any alcohol did you have each day? | Monday └─┴─┘  Tuesday └─┴─┘  Wednesday └─┴─┘  Thursday └─┴─┘  Friday └─┴─┘  Saturday └─┴─┘  Sunday └─┴─┘  77. Don’t know. |

***3). Diet***

The next questions ask about the fruits and vegetables that you usually eat. I have a nutrition card here that shows you some examples of local fruits and vegetables. Each picture represents the size of a serving. As you answer these questions please think of a typical week in the last year.

| **Code** | **Questions** | **Response** |
| --- | --- | --- |
| Q 28 | In a typical week, how many days do you eat fruit?  *(USE SHOWCARD)* | Number of days └─┴─┘if 0 Go to Q 30  77. Don’t know |
| Q 29 | How many servings of fruits do you eat on one of those days?  *(USE SHOWCARD)* | Number of servings └─┴─┘  77. Don’t know |
| Q 30 | In a typical week, on how many days do you eat vegetables?  *(USE SHOWCARD)* | Number of days └─┴─┘  77. Don’t know |
| Q 31 | How many servings of vegetables do you eat on one of those days?  *(USE SHOWCARD)* | Number of days └─┴─┘  77. Don’t know |
| Q 32 | On average, how many meals per week do you eat that were not prepared at a home? By meal, I mean breakfast, lunch and dinner. | Number of days └─┴─┘  77. Don’t know |
| Q 33 | What types of oils or fat is most often used for meal preparation in your household? (only one answer) | 1. Vegetable oil 2. Lard 3. Butter 4. Margarine 5. Others (specify):……………. 6. None in particular 7. None used   77. Don’t know |
| Q 34 | How often do you add salt or a salty sauce such as soya sauce or fish sauce to your food right before you eat it or as you are eating it?  (SELECT ONLY ONE)  (USE SHOWCARD) | 1. Always 2. Often 3. Sometimes 4. Rarely 5. Never   77. Don’t know |
| Q 35 | How often do you eat processed food high in salt? By processed food high in salt, I mean foods that have been altered from their natural state, such as salted fish, salted meat, salted eggs instant noodle, fermented fish/prahok, paok, mam, kapi, packaged salty snacks, canned salty food including pickles and preserves, salty food prepared at a fast food restaurant, cheese, bacon and processed meat.  (USE SHOWCARD) | 1. Always 2. Often 3. Sometimes 4. Rarely 5. Never   77. Don’t know |

***4). Physical Activity***

Next, I am going to ask you about the time you spend doing different types of physical activity in a typical week. Please answer these questions even if you do not consider yourself to be a physically active person.

Think first about the time you spend doing work. Think of work as the things that you have to do such as paid or unpaid work, study/training, household chores, harvesting food/crops, planting crops, gardening, walking/cycling to rice field, ploughing, digging, construction work, fishing or hunting for food, seeking employment. In answering the following questions 'vigorous-intensity activities' are activities that require hard physical effort and cause large increases in breathing or heart rate, 'moderate-intensity activities' are activities that require moderate physical effort and cause small increases in breathing or heart rate.

| **Work** | | |
| --- | --- | --- |
| **Code** | **Questions** | **Response** |
| Q 36 | Does your work involve vigorous-intensity activity that causes large increases in breathing or heart rate like ploughing, digging, lifting heavy loads, or construction work for at least 10 minutes continuously?  (USE SHOWCARD) | 1. Yes 2. No (Go to Q 38) |
| Q 37 | In a typical week, on how many days do you do vigorous-intensity activities as part of your work? | Number of days └─┴─┘ |
| Q 38 | How much time do you spend doing vigorous-intensity activities at work on a typical day? | Hours : minutes └─┴─┘:└─┴─┘ |
| Q 39 | Does your work involve moderate-intensity activity that causes small increases in breathing or heart rate such as brisk walking, planting or harvesting crops, cleaning, washing by hand, or cycling for at least 10 minutes continuously?  (USE SHOWCARD) | 1. Yes 2. No (Go to Q 41) |
| Q 40 | In a typical week, on how many days do you do moderate-intensity activities as part of your work? | Number of days └─┴─┘ |
| Q 41 | How much time do you spend doing moderate-intensity activities at work on a typical day? | Hours : minutes └─┴─┘:└─┴─┘ |

| **Travel to and from Places** | | |
| --- | --- | --- |
| Other than activities that you have already mentioned, I would like to ask you about the way you travel to and from places. For example, to work, for shopping, to the field, to market, to church, funerals, gatherings. | | |
| Q 42 | Do you walk or use a bicycle (pedal cycle) for at least 10 minutes continuously to get to and from places? | 1. Yes 2. No (Go to Q 45) |
| Q 43 | In a typical week, on how many days do you walk or bicycle for at least 10 minutes continuously to get to and from places? | Number of days └─┴─┘ |
| Q 44 | How much time do you spend walking or bicycling for travel on a typical day? | Hours : minutes └─┴─┘:└─┴─┘ |

| **Recreational Activities** | | |
| --- | --- | --- |
| The next questions exclude the work and transport activities that you have already mentioned.  Now I would like to ask you about sports, fitness and recreational activities (leisure). | | |
| **Code** | **Questions** | **Response** |
| Q 45 | Do you do any vigorous-intensity sports, fitness or recreational (leisure) activities that cause large increases in breathing or heart rate like [running or football] for at least 10 minutes continuously?  (USE SHOWCARD) | 1. Yes 2. No (Go to Q 48) |
| Q 46 | In a typical week, on how many days do you do vigorous-intensity sports, fitness or recreational (leisure) activities? | Number of days └─┴─┘ |
| Q 47 | How much time do you spend doing vigorous-intensity sports, fitness or recreational activities on a typical day? | Hours : minutes └─┴─┘:└─┴─┘ |
| Q 48 | Do you do any moderate-intensity sports, fitness or recreational (leisure) activities that cause a small increase in breathing or heart rate such as, [cycling, swimming, or volleyball] for at least 10 minutes continuously?  (USE SHOWCARD) | 1. Yes 2. No (Go to Q 51) |
| Q 49 | In a typical week, on how many days do you do moderate-intensity sports, fitness or recreational (leisure) activities? | Number of days └─┴─┘ |
| Q 50 | How much time do you spend doing moderate-intensity sports, fitness or recreational (leisure) activities on a typical day? | Hours : minutes └─┴─┘:└─┴─┘ |

| **Sedentary Behavior** | | |
| --- | --- | --- |
| The following question is about sitting or reclining. Think back over the past 7 days, to the time spent at work, at home, in leisure, including time spent sitting at a desk, visiting friends, reading, or watching television, but do not include time spent sleeping. | | |
| **Code** | **Questions** | **Response** |
| Q 51 | Over the past 7 days, how much time did you spend sitting or reclining on a typical day? | Hours : minutes └─┴─┘:└─┴─┘ |

***5). Expanded Sections***

| **History of High Blood Pressure** | | |
| --- | --- | --- |
| **Code** | **Questions** | **Response** |
| Q 52 | Have you ever had your blood pressure measured by a doctor or other health worker? | 1. Yes  2. No (Go to Q 57) |
| Q 53 | Have you ever been told by a doctor or other health worker that you have raised blood pressure or hypertension during the last 12 months? | 1. Yes  2. No (Go to Q 57) |
| Q 54 | Have you been taking any drugs prescribed by a doctor or health worker in the past 2 weeks for elevated blood pressure? | 1. Yes 2. No |
| Q 55 | Have you ever consulted traditional healer for elevated blood pressure or hypertension? | 1. Yes 2. No |
| Q 56 | Are you currently taking any herbal or traditional remedy for your high blood pressure? | 1. Yes 2. No |

| **History of Diabetes** | | |
| --- | --- | --- |
| **Code** | **Questions** | 1. **Response** |
| Q 57 | Have you ever had your blood sugar measured by a doctor or other health worker? | 1. Yes 2. No (Go to Q 63) |
| Q 58 | Have you ever been diagnosed by a doctor or other health worker that you have raised blood sugar or diabetes? | 1. Yes 2. No |
| Q 59 | Have you been told in the past 12 months? | 1. Yes 2. No |
| Q 60 | In the past two weeks, have you taken any drugs (medication) for diabetes prescribed by a doctor or other health worker? | 1. Yes 2. No |
| Q 61 | Are you currently taking insulin for diabetes prescribed by a doctor or other health worker? | 1. Yes 2. No |
| Q 62 | Are you currently taking any herbal or traditional remedy for your diabetes? | 1. Yes 2. No |

| **History of Raised Total Cholesterol** | | |
| --- | --- | --- |
| **Code** | **Questions** | **Response** |
| Q 63 | Have you ever had your cholesterol (fat levels in your blood) measured by a doctor or other health worker? | 1. Yes  2. No (Go to Q 68) |
| Q 64 | Have you ever been told by a doctor or other health worker that you have raised cholesterol? | 1. Yes  2. No (Go to Q 68) |
| Q 65 | Have you been told in the past 12 months? | 1. Yes  2. No |
| Q 66 | In the past two weeks, have you taken any oral treatment (medication) for raised total cholesterol prescribed by a doctor or other health worker? | 1. Yes  2. No |
| Q 67 | Are you currently taking any herbal or traditional remedy for your raised cholesterol? | 1. Yes  2. No |

| **History of Lifestyle Advice** | | |
| --- | --- | --- |
| Q 68: During the past three years, has a doctor or other health worker advised you to do any of the following? | | |
| **Code** | **Questions** | **Response** |
| Q 68.1 | Quit using tobacco or don’t start | 1. Yes  2. No |
| Q 68.2 | Reduce salt in your diet | 1. Yes  2. No |
| Q 68.3 | Eat at least five servings of fruit and/or vegetables each day | 1. Yes  2. No |
| Q 68.4 | Reduce fat in your diet | 1. Yes  2. No |
| Q 68.5 | Start or do more physical activity | 1. Yes  2. No |
| Q 68.6 | Maintain a healthy body weight or lose weight | 1. Yes  2. No |

| **Family History and OC Use** | | |
| --- | --- | --- |
| **Code** | **Questions** | **Response** |
| Q 69 | Has any of your family member been diagnosed with diabetes? | 1. Yes  2. No  77. Don’t know |
| Q 70 | Has any of your family member been diagnosed with hyperlipidemia? | 1. Yes  2. No  77. Don’t know |
| Q 71 | Has any of your family member been diagnosed with hypertension? | 1. Yes  2. No  77. Don’t know |
| Q 72 | Has any of your family member been diagnosed with heart attack or chest pain from heart disease (angina) or stroke? | 1. Yes  2. No  77. Don’t know |
| Q 73 | Are you currently using oral contraceptives?  *(FEMALE PARTICIPANTS ONLY)* | 1. Yes  2. No |
| Q 74 | Do you know if you were born prematurely or on term? | 1. Prematurely born  2. On term  77. Don’t know |

**STEPS 2: Physical Measurements**

| Interviewer ID | | └─┴─┘ |
| --- | --- | --- |
| **Height, Weight and Waist and Hip Circumference** | | |
| **Code** | **Question** | **Response** |
| Q 75 | Device ID for height and weight | Height └─┴─┘  Weight └─┴─┘ |
| Q 76 | Height measured to the nearest 0.1cm | Height (cm)  └─┴─┴ . ┴─┘ |
| Q 77 | Weight measured to the nearest 0.1 kg | Weight (kg)  └─┴─┴ . ┴─┘ |
| Q 78 | Device ID for waist and hip | └─┴─┘ |
| Q 79 | Waist circumference (to the nearest 0.1cm) | └─┴─┴ . ┴─┘ cm |
| Q 80 | Hip circumference (to the nearest 0.1cm) | └─┴─┴ . ┴─┘ cm |
| **Blood Pressure (Reading to be Five Minutes Apart)** | | |
| Q 81 | Device ID for blood pressure | └─┴─┘ |
| Q 82 | Reading 1 | Systolic BP └─┴─┴─┘ mmHg  Diastolic BP └─┴─┴─┘ mmHg |
| Q 83 | Reading 2 | Systolic BP └─┴─┴─┘ mmHg  Diastolic BP └─┴─┴─┘ mmHg |
| Q 84 | Reading 3 | Systolic BP └─┴─┴─┘ mmHg  Diastolic BP └─┴─┴─┘ mmHg |
| Q 85 | During the past two weeks, have you been treated for raised blood pressure with drugs (medication) prescribed by a doctor or other health worker? |  |

**STEPS 3: Biochemical Measurements**

| Interviewer ID | | └─┴─┘ |
| --- | --- | --- |
| **Code** | **Question** | **Response** |
| **Blood Glucose** | | |
| Q 86 | During the last 12 hours have you had anything to eat or drink, other than water? | 1. Yes 2. No |
| Q 87 | Device ID | └─┴─┘ |
| Q 88 | Time of day blood specimen taken (24h clock) | Hours : minutes └─┴─┘:└─┴─┘ |
| Q 89 | Fasting blood glucose | └─┴─┴. └─┴─┴ mmol/l or mg/dl |
| **Blood Lipids** | | |
| Q 90 | Device ID | └─┴─┘ |
| Q 91 | Total Cholesterol | └─┴─┴. └─┴─┴ mmol/l or mg/dl |
| END for participants at VCCT sites (those who come for HIV testing)  **THANK YOU FOR PARTICIPATING IN THIS STUDY** | | |

**PART II: HIV and ART Inventory**

| Only participants at ART sites (those who come for ART) answer Part II | | |
| --- | --- | --- |
| Interviewer ID | | └─┴─┘ |
| **Code** | **Questions** | **Response** |
| Q 92 | How long have you been living with HIV?  *(CHOOSE ONLY ONE TO ANSWER, NOT BOTH)* | Number of months └─┴─┘  Number of years └─┴─┘ |
| Q 93 | How long have you been on ART?  *(CHOOSE ONLY ONE TO ANSWER, NOT BOTH)* | Number of months └─┴─┘  Number of years └─┴─┘ |
| Q 94 | Current ART regimen  *(LOOK IN FOLLOW-UP BOOKLET)* | ART regimen: …………………………………….  Starting date:  └─┴─┘└─┴─┘└─┴─┴─┘ |
| Q 95 | Previous ART regimen  *(LOOK IN FOLLOW-UP BOOKLET)* | ART regimen:  ……………………………………………………  Starting date:  └─┴─┘└─┴─┘└─┴─┴─┘ |
| Q 96 | Are you currently on any medication to prevent or to treat opportunistic infections? | 1. Yes  2. No |
| Q 97 | Are you currently taking tuberculosis treatment? | 1. Yes  2. No |
| Q 98 | During the past 6 months, have you received blood test for CD4 count? | 1. Yes  2. No (End) |
| Q 99 | What was your CD4 count when you received the most recent blood test? | CD4 count └─┴─┴─┘  (00 if do not know) |
| **THANK YOU FOR PARTICIPATING IN THIS STUDY** | | |
