# Supplementary material for: Differences in prevalence and risk factors of non-communicable diseases between young people living with HIV (YLWH) and young general population in Cambodia
Source: PLoS One. 2022 Jun 21;17(6):e0269989. doi: 10.1371/journal.pone.0269989 (PMC9212152; doi:10.1371/journal.pone.0269989)
Supplement: S2 File — (DOCX) [file pone.0269989.s002.docx]

កម្រងសំណួរ

សម្រាប់ការតាមដានកត្តាគ្រោះថ្នាក់របស់ជំងឺមិនឆ្លង

ព្រះរាជាណាចក្រកម្ពុជា ២០១៩

សំរាប់ពត៌មានបន្ថែម សូមទំនាក់ទំនងមកអ្នកដឹកនាំគម្រោងស្រាវជ្រាវ ឈ្មោះ៖

សៀង កិន្នរី (សាកលវិទ្យាល័យនៃរដ្ឋកាលីហ្វ័រនីញ៉ា ក្រុងឡសអេនជឺឡេស នៅសហរដ្ឋអាមេរិច)

លេខទូរស័ព្ទ៖ ០៩៩ ៧១៤៧៧៩

អ៊ីមែល៖ [seang.kennarey@gmail.com](mailto:seang.kennarey@gmail.com) ឬ kennareyseang@ucla.edu

ព័ត៌មានសម្រាប់ការអង្កេត

| **ទីកន្លែងធ្វើអង្កេត និងកាលបរិច្ឆេទ** | |
| --- | --- |
| លេខកូដសម្គាល់ខ្លួនរបស់**អ្នកចូលរួមការអង្កេត** | └─┴─┴─┘ |
| ឈ្មោះមណ្ឌល | ………………………………….. |
| កាលបរិច្ឆេទនៃការសម្ភាស | ថ្ងៃ ខែ ឆ្នាំ  └─┴─┘└─┴─┘└─┴─┴─┘ |
| លេខកូដសម្គាល់ខ្លួនរបស់អ្នកសម្ភាស | └─┴─┘ |
| **ការយល់ព្រមចូលរួមការអង្កេត** | |
| អ្នកចូលរួមបានអាន និងទទួលលិខិតយល់ព្រមចូលរួមការអង្កេត | 1. បាទ/ចាស 2. ទេ (បើ ទេ សូមបញ្ចប់ការសម្ភាស) |
| ម៉ោងធ្វើសម្ភាស (គិតជា 24 ម៉ោង) | ម៉ោង:នាទី └─┴─┘:└─┴─┘ |
| អ្នកត្រួតពិនិត្យ  កាលបរិច្ឆេទនៃការត្រួតពិនិត្យ | ឈ្មោះ……………………………………………  ថ្ងៃ ខែ ឆ្នាំ  └─┴─┘└─┴─┘└─┴─┴─┘ |

ស្តេប ១**:** ព័ត៌មានប្រជាសាស្រ្ត

| កូដ | សំណួរ | ចម្លើយ |
| --- | --- | --- |
| Q 1 | ភេទ (កត់ត្រាចម្លើយ ប្រុស/ស្រី តាមការមើលឃើញ) | 1. ប្រុស 2. ស្រី |
| Q 2 | តើអ្នកកើតក្នុង ខែ ឆ្នាំណា? | ខែ ឆ្នាំ (រំលងទៅ Q 4)  └─┴─┘└─┴─┴─┘  មិនដឹង សរសេរលេខ 77 |
| Q 3 | តើអ្នកមានអាយុប៉ុន្មាន? | └─┴─┘ឆ្នាំ |
| Q 4 | តើអ្នកមានស្ថានភាពគ្រួសារបែបណា? | 1. នៅលីវ 2. រៀបការរស់នៅជាមួយគ្នា 3. រស់នៅបែកគ្នា 4. លែងលះគ្នា 5. មេម៉ាយ/ពោះម៉ាយ 6. រស់នៅជាមួយគ្នាដោយមិនបានរៀបការ   88. បដិសេធមិនឆ្លើយ |
| Q 5 | តើអ្នករៀនបាន**ខ្ពស់បំផុត**ដល់កម្រិតណា? | 1. មិនបានចូលរៀននៅសាលា 2. បញ្ចប់ថ្នាក់បថមសិក្សា 3. បញ្ចប់ថ្នាក់មធ្យមសិក្សា 4. បញ្ចប់ថ្នាក់វិទ្យាល័យ 5. បញ្ចប់មហាវិទ្យាល័យ (បរិញ្ញាប័ត្ររង) 6. មហាវិទ្យាល័យ (បរិញ្ញាប័ត្រជាន់ខ្ពស់) និង ថ្នាក់ក្រោយឧត្តមសិក្សា   88. បដិសេធមិនឆ្លើយ |
| Q 6 | តើរយៈពេល ១២ខែ កន្លងមកនេះ អ្នកមានមុខរបរ**ចម្បងជា**អ្វី? | 1. មន្រ្តីរាជការ 2. មិនមែនមន្រ្តី/បុគ្កលិករាជការ 3. ប្រកបរបដោយខ្លួនឯង 4. សិស្ស/និស្សិត 5. មេផ្ទះ 6. ចូលនិវត្តន៍ 7. គ្មានការងារធ្វើ 8. ផ្សេងៗ………………………   88. បដិសេធមិនឆ្លើយ |
| Q 7 | តើមានសមាជិកគ្រួសារដែលមានអាយុចាប់ពី 18ឆ្នាំ ប៉ុន្មាននាក់ ដែលរស់នៅក្នុងផ្ទះនេះ រាប់ទាំងរូបអ្នកផង? | └─┴─┘នាក់ |
| Q 8 | កន្លងទៅ តើអ្នកអាចប្រាប់បានទេ ថាប្រាក់ចំណូល របស់គ្រួសារ ជាមធ្យមប៉ុន្មាន? *(កត់ត្រាចម្លើយតែមួយគត់ មិនមែនកត់ត្រាទាំងបីនោះទេ)* | ក្នុងមួយអាទិត្យ:…………………(រំលងទៅ Q10)  ឬ ក្នុងមួយខែ:………………….. (រំលងទៅ Q10)  ឬក្នុងមួយឆ្នាំ:……………………… (រំលងទៅ Q10)  88. បដិសេធមិនឆ្លើយ |
| Q 9 | បើអ្នកមិនដឹងអំពីប្រាក់ចំណូលប្រចាំឆ្នាំជាក់លាក់របស់គ្រួសារ តើអ្នកអាចប៉ាន់ស្មានបាននូវចំណូលទាំងនេះបានទេ?  [*ប្រាក់ចំណូលគិតជាលុយរៀល*]  *(អានជម្រើសនីមួយៗឲ្យស្តាប់)* | 1. តិចជាង ឬស្មើ មួយលាន និងប្រាំមឺន រៀល (≤1 050 000 រៀល 2. លើសពី មួយលាន និងប្រាំមឺន រៀល ទៅ បួនលាន រៀល (1 050 000 រៀល ទៅ 4 000 000 រៀល) 3. លើសពីបួនលានរៀល ទៅ ដប់ប្រាំពីរលាន និង ប្រាំសែនរៀល ( 4 000 000 រៀល ទៅ 17 500 000 រៀល) 4. លើសពី ដប់ប្រាំពីរលាន និងប្រាំសែនរៀល ទៅ ម្ភៃមួយលាន ប៉ែតសិបប្រាំពីរមឺន និងប្រាំពាន់រៀល (17 500 000 រៀល ទៅ 21 875 000 រៀល) 5. លើសពីម្ភៃមួយលាន ប៉ែតសិបប្រាំពីរមឺន និងប្រាំពាន់រៀល (≥21 875 000 រៀល)   77. មិនដឹង  88. បដិសេធមិនឆ្លើយ |

ស្តេប ១**:** ការវាស់វែងឥរិយាបថប្រឈម

*១) ការប្រើថ្នាំជក់*

ឥឡូវនេះ ខ្ញុំនឹងសាកសួរអ្នកនូវសំណួរមួយចំនួនពីការប្រើថ្នាំជក់។

| កូដ | សំណួរ | ចម្លើយ |
| --- | --- | --- |
| Q 10 | តើសព្វថ្ងៃនេះ អ្នកកំពុងប្រើ**ថ្នាំជក់** ដូចជាបារី ស៊ីហ្គា ឬជក់ខ្សៀដែរឬទេ?  *(*សូមបង្ហាញផ្ទាំងរូបភាព) | 1. បាទ/ចាស 2. ទេ (រំលងទៅ Q 14) |
| Q 11 | តើអ្នកកំពុងជក់បារី**រាល់ថ្ងៃ**ដែរឬទេ? | 1. បាទ/ចាស 2. ទេ |
| Q 12 | តើអ្នកមានអាយុប៉ុន្មាន នៅពេលដែលអ្នក  **ចាប់ផ្តើមជក់បារីជាលើកដំបូង**? | └─┴─┘ឆ្នាំ  99. មិនចាំ |
| Q 13 | ជាមធ្យម **ក្នុងមួយថ្ងៃ/ក្នុងមួយអាទិត្យ** តើអ្នកជក់បារី ដូចរៀបរាប់ជូននេះ ប៉ុន្មានដើម?  *(ប្រសិនបើ​តិចជាងប្រចាំថ្ងៃ, សូមកត់ត្រាចំនួនជាប្រចាំសប្តាហ៏)*  *(*សូមកត់ត្រាប្រភេទបារី នីមួយៗ និងបង្ហាញផ្ទាំងរូបភាព) | ក្នុង១ថ្ងៃ ក្នុង១អាទិត្យ  1. បារីកញ្ចប់ └─┴─┴─┘..└─┴─┴─┘  2. បារីមូរដៃ └─┴─┴─┘..└─┴─┴─┘  3. ខ្សៀរដែល  ដាក់ថ្នាំជក់ពេញ └─┴─┴─┘..└─┴─┴─┘  4. ស៊ីហ្គា បារី  មូរស្លឹកសង្កែ  ស៊ីស្សា └─┴─┴─┘..└─┴─┴─┘  5. ផ្សេងៗ └─┴─┴─┘..└─┴─┴─┘  ផ្សេងៗ (សូមបញ្ជាក់):…………………………….  77. មិនដឹង |
| Q 14 | កាលពីមុន តើអ្នក**ធ្លាប់ជាអ្នកជក់បារីដែរឬទេ**? | 1. បាទ/ចាស 2. ទេ (រំលងទៅ Q 16) |
| Q 15 | បើជាអ្នកជក់បារី តើអ្នកជក់រៀងរាល់ថ្ងៃដែរឬទេ? | 1. បាទ/ចាស 2. ទេ   99. មិនចាំ |
| Q 16 | តើអ្នក**កំពុងប្រើប្រាស់ផលិតផលថ្នាំជក់ (មិនមែនជក់បារីទេ)** ដូចជាចុកថ្នាំ ឬចុកថ្នាំជក់លាយជាមួយស្លាម្លូ ដែរឬទេ?  *(សូមបង្ហាញផ្ទាំងរូបភាព)* | 1. បាទ/ចាស 2. ទេ (រំលងទៅ Q 18) |
| Q 17 | ជាមធ្យម ក្នុងមួយថ្ងៃ/អាទិត្យ តើអ្នកប្រើប្រាស់ផលិតផលនេះប៉ុន្មានដង ….  *(បើមិនមែនរាល់ថ្ងៃ សូមកត់ត្រាចម្លើយសម្រាប់មួយ អាទិត្យ)* | ក្នុង១ថ្ងៃ ក្នុង១អាទិត្យ  1. ចុកថ្នាំជក់ └─┴─┴─┘..└─┴─┴─┘  2. ចុកថ្នាំជក់លាយ  ជាមួយស្លាម្លូ └─┴─┴─┘..└─┴─┴─┘  3. ផ្សេងៗ └─┴─┴─┘..└─┴─┴─┘  ផ្សេងៗ (សូមបញ្ជាក់):……………………………. |
| Q 18 | ក្នុងរយៈពេល ៣០ថ្ងៃ កន្លងមកនេះ តើមានអ្នកណាម្នាក់ជក់បារី**នៅក្នុងផ្ទះរបស់អ្នក**ដែរឬទេ? | 1. មាន 2. មិនមាន |

*២) ការទទួលទានគ្រឿងស្រវឹង*

សំណួរបន្ទាប់មកទៀតនេះ យើងនឹងសាកសួរអ្នកពីការទទួលទានគ្រឿងស្រវឹង។

| កូដ | សំណួរ | ចម្លើយ |
| --- | --- | --- |
| Q 19 | តើអ្នក**ធ្លាប់**ទទួលទានគ្រឿងស្រវឹងដូចជា ស្រាបៀរ ស្រាដែលធ្វើពីផ្លែឈើ ទឹកត្នោតជូរ ស្រា ស្រាស និងស្រាថ្នាំដែរឬទេ?  *(*សូមបង្ហាញផ្ទាំងរូបភាព ឬឲ្យឧទាហរណ៍) | 1. បាទ/ចាស 2. ទេ (រំលងទៅ Q 22) |
| Q 20 | **ក្នុងរយៈពេល ១២ខែ កន្លងមកនេះ** តើអ្នកបានទទួលទានគ្រឿងស្រវឹងយ់ាងហោចណាស់ ១កម្រិតស្តង់ដា ដែរឬទេ? | 1. បាទ/ចាស 2. ទេ (រំលងទៅ Q 22) |
| Q 21 | ក្នុងរយៈពេល ១២ខែ កន្លងមកនេះ តើអ្នកធ្លាប់បានទទួលទាន គ្រឿងស្រវឹងយ៉ាងហោចណាស់ ១កម្រិត  **ញឹកញាប់ប៉ុន្មាន**?  *(*សូមអានចម្លើយ និងបង្ហាញផ្ទាំងរូបភាព*) (USE SHOWCARD)* | 1. រាល់ថ្ងៃ 2. ៥-៦ ថ្ងៃ ក្នុង ១អាទិត្យ 3. ៣-៤ ថ្ងៃ ក្នុង ១អាទិត្យ 4. ១-២ ថ្ងៃ ក្នុង ១អាទិត្យ 5. ១-៣ ថ្ងៃ ក្នុង ១ខែ 6. តិចជាងម្តង ក្នុង ១ខែ |
| Q 22 | ក្នុង**រយៈពេល​ ៣០ថ្ងៃ កន្លងមកនេះ** តើអ្នកធ្លាប់បានទទួលទានគ្រឿងស្រវឹងយ៉ាងហោចណាស់ ១កម្រិតស្តង់ដា ដែរឬទេ? | 1. បាទ/ចាស 2. ទេ (រំលងទៅ Q 27) |
| Q 23 | ក្នុងរយៈពេល​ ៣០ថ្ងៃ កន្លងមកនេះ តើមាន**ចំនួនប៉ុន្មានពេល**ដែលអ្នកទទួលទាន យ៉ាងហោចណាស់ គ្រឿងស្រវឹង១កម្រិតស្តង់ដា? | ចំនួន └─┴─┘  77. មិនដឹង |
| Q 24 | ក្នុងរយៈពេល​ ៣០ថ្ងៃ កន្លងមកនេះ ពេលអ្នកទទួលទានគ្រឿង **ជាមធ្យម តើអ្នកទទួលទានប៉ុន្មានកម្រិតស្តង់ដា**ក្នុងមួយពេល?  *(*សូមបង្ហាញផ្ទាំងរូបភាព) | ចំនួន └─┴─┘  77. មិនដឹង |
| Q 25 | ក្នុងរយៈពេល​ ៣០​ថ្ងៃ កន្លងមកនេះ តើចំនួនកម្រិតស្តង់ដាគ្រឿងស្រវឹង**ច្រើនជាងគេ** មានប៉ុន្មានដែលអ្នកបានទទួលទាន ក្នុងមួយពេល រាប់បញ្ចូលគ្រឿងស្រវឹងគ្រប់ប្រភេទ? | ចំនួនច្រើនជាងគេ └─┴─┘  77. មិនដឹង |
| Q 26 | ក្នុងរយៈពេល​ ៣០ថ្ងៃ កន្លងមកនេះ មានប៉ុន្មានដងដែលអ្នកទទួលទានគ្រឿងស្រវឹង **៦ កម្រិត**  **ស្តង់ដា ឬលើសពីនេះ** ក្នុងមួយពេលៗ? | ចំនួនដង └─┴─┘  77. មិនដឹង |
| Q 27 | **ក្នុងរយៈពេល ៧ថ្ងៃ កន្លងមកនេះ** តើក្នុងមួយថ្ងៃៗ អ្នកទទួលទានគ្រឿងស្រវឹងប៉ុន្មានកម្រិតស្តង់ដា?  *(*សូមបង្ហាញផ្ទាំងរូបភាព*)* | ថ្ងៃច័ន្ទ └─┴─┘  ថ្ងៃអង្គារ └─┴─┘  ថ្ងៃពុធ └─┴─┘  ថ្ងៃព្រហស្សត៍ └─┴─┘  ថ្ងៃសុក្រ └─┴─┘  ថ្ងៃសៅរ៍ └─┴─┘  ថ្ងៃអាទិត្យ └─┴─┘  77. មិនដឹង |

*៣). របបអាហារ*

សំណួរបន្ទាប់ យើងនឹងសាកសួរអំពីបន្លែ និងផ្លែឈើដែលអ្នកបរិភោគជាធម្មតា។ យើងខ្ញុំមានផ្ទាំងរូបភាពអំពីរបបអាហារ នៅទីនេះដើម្បីបង្ហាញអ្នកជាឧទាហរណ៍ ពីឈ្មោះបន្លែ និងផ្លែឈើ មួយចំនួន។ រូបភាពនីមួយៗតំណាងឲ្យរង្វាល់ដែលត្រូវបរិភោគ។ នៅពេលអ្នកឆ្លើយនឹងសំណួរនេះ ចូរអ្នកគិតដល់អាទិត្យណាមួយកាលពីឆ្នាំកន្លងទៅ។

| កូដ | សំណួរ | ចម្លើយ |
| --- | --- | --- |
| Q 28 | នៅក្នុងមួយអាទិត្យ តើអ្នក**បរិភោគផ្លែឈើ** ប៉ុន្មានថ្ងៃ?  *(*សូមបង្ហាញផ្ទាំងរូបភាព*)* | ចំនួនថ្ងៃ └─┴─┘បើ សូន្យរំលងទៅ Q 30  77. មិនដឹង |
| Q 29 | តើអ្នកបរិភោគផ្លែឈើ**ប៉ុន្មានរង្វាល់** នៅក្នុងមួយថ្ងៃ ទាំងនោះ?  (*សូមបង្ហាញផ្ទាំងរូបភាព)* | រង្វាល់ └─┴─┘  77. មិនដឹង |
| Q 30 | នៅក្នុងមួយអាទិត្យ តើអ្នក**បរិភោគបន្លែ** ប៉ុន្មានថ្ងៃ?  *(*សូមបង្ហាញផ្ទាំងរូបភាព*)* | ចំនួនថ្ងៃ └─┴─┘  77. មិនដឹង |
| Q 31 | តើអ្នកបរិភោគបន្លែ**ប៉ុន្មានរង្វាល់** នៅក្នុងមួយថ្ងៃ ទាំងនោះ?  (*សូមបង្ហាញផ្ទាំងរូបភាព)* | រង្វាល់ └─┴─┘  77. មិនដឹង |
| Q 32 | ជាមធ្យម ក្នុងមួយអាទិត្យ តើអ្នកបរិភោគអាហារពេលព្រឹក ថ្ងៃត្រង់ និងពេលល្ងាចដែលចម្អិននៅខាងក្រៅផ្ទះប៉ុន្មានដង? | ចំនួនដង └─┴─┘  77. មិនដឹង |
| Q 33 | តើប្រេង ឬខ្លាញ់ប្រភេទណា ដែលអ្នកប្រើញឹកញាប់សម្រាប់ចម្អិនអាហារនៅផ្ទះរបស់អ្នក?  *(*សូមបង្ហាញផ្ទាំងរូបភាព*)* | 1. ប្រេងរុក្ខជាតិ 2. ខ្លាញ់សត្វ 3. ប៊ឺរ 4. ម៉ាហ្គារីន 5. ផ្សេងៗ………………………. 6. មិនច្បាស់លាស់ណាមួយ 7. មិនប្រើអ្វីទាំងអស់   77. មិនដឹង |
| Q 34 | **មុននឹងបរិភោគអាហារ ឬកំពុងបរិភោគអាហារ តើអ្នកតែងតែ**ថែមអំបិល ឬទឹកជ្រលក់ដែលមានរសជាតិប្រៃដូចជាទឹកស៊ីអ៊ីវ  ទឹកត្រី**ទៅក្នុងអាហារនោះ ញឹកញាប់ប៉ុណ្ណា?**  **(*ជ្រើសរើសចំលើយបានតែមួយគត់)***  (*សូមបង្ហាញផ្ទាំងរូបភាព)* | 1. ជានិច្ចកាល 2. ញឹកញាប់ 3. ម្តងម្កាល 4. កម្រណាស់ 5. មិនដែលទាល់តែសោះ   77. មិនដឹង |
| Q 35 | **តើអ្នកបរិភោគចំណី**អាហារកែច្នៃមានបរិមាណអំបិលច្រើន **ញឹកញាប់ប៉ុណ្ណា? អាហារកែច្នៃមានបរិមាណអំបិលច្រើនមានន័យថាម្ហូបដែលគេបានកែច្នៃឲ្យខុសពីសភាពធម្មជាតិ ដូចជាត្រីងៀត ពងទាប្រៃ មីកញ្ចប់ ប្រហុក ផ្អក មាំ កាពិ នំកញ្ចប់ដែលមានរសជាតិប្រៃ អាហារកំប៉ុងដែលមានជាតិប្រៃដូចជាត្រសក់ជ្រក់ និងម្ហូបអាហារដែលមានរសជាតិប្រៃ ធ្វើនៅភោជនីដ្ធានទាន់ចិត្ត​​ ឈីស​ បាខុន និងសាច់កែឆ្នៃ** | 1. ជានិច្ចកាល 2. ញឹកញាប់ 3. ម្តងម្កាល 4. កម្រណាស់ 5. មិនដែលទាល់តែសោះ   77. មិនដឹង |

*៤) កាយវប្បកម្ម*

បន្ទាប់មកទៀតនេះ យើងនឹងសាកសួរអ្នកអំពីរយៈពេលដែលអ្នកចំណាយសម្រាប់ធ្វើកាយវប្បកម្ម នៅក្នុងអាទិត្យដ៏ជាក់លាក់ណាមួយ សូមឆ្លើយនូវសំណួរខាងក្រោមនេះទោះបីជាអ្នកគិតថាខ្លួនឯងមិនមែនជាអ្នកសកម្មខាងកាយវប្បកម្មក៏ដោយ។

ដំបូងគិតអំពីពេលវេលាដែលអ្នកចំណាយនៅពេលធ្វើការងារជាមុនសិន។ ចូរគិតថាការងារ គឺជាអ្វីដែលអ្នកត្រូវធ្វើ ដូចជា (បានប្រាក់កម្រៃ ឬមិនបានក៏ដោយ ការសិក្សា/បណ្តុះបណ្តាល ការងារផ្ទះ ប្រមូលកសិផល ធ្វើចំការដាំដំណាំ ដើរ ជិះកង់ទៅវាលស្រែ ភ្ជួររាស់ដី កាប់គាស់ដី ការងារសំណង់ ស្ទូចត្រី ឬបរបាញ់សត្វដើម្បីយកមកធ្វើជាម្ហូបអាហារ ការដើរស្វែងរកការងារធ្វើ។ ដើម្បីឆ្លើយសំណួរខាងក្រោម “សកម្មភាពខ្លាំងក្លា” ជាសកម្មភាពទាំងឡាយណាដែលតម្រូវឲ្យមានការបញ្ចេញកម្លាំងពលកម្មខ្លាំង ធ្វើឲ្យចង្វាក់ដង្ហើម និងបេះដូងដើរញាប់ ខ្លាំងជាងធម្មតា។

“សកម្មភាពមធ្យម” ជាសកម្មភាពទាំងឡាយណាដែលតម្រូវឲ្យមានការបញ្ចេញកម្លាំងពលកម្មមធ្យម ធ្វើឲ្យចង្វាក់ដង្ហើម និងបេះដូងដើរញាប់ល្មម។

| **នៅកន្លែងធ្វើការ** | | |
| --- | --- | --- |
| កូដ | សំណួរ | ចម្លើយ |
| Q 36 | តើការងារដែលអ្នកធ្វើមានសកម្មភាពខ្លាំងក្លា អាចឲ្យអ្នកហត់ដង្ហក់ ឬបេះដូងដើរញាប់ ដូចជាភ្ជួររាស់ដី កាប់គាស់ដី លីសែងវត្ថុធ្ងន់ៗ ឬការងារសំណង់ យ៉ាងតិច ១០នាទីជាប់ៗគ្នាដែរឬទេ?  *(សូមបង្ហាញផ្ទាំងរូបភាព)* | 1. បាទ/ចាស 2. ទេ (រំលងទៅ Q 38) |
| Q 37 | តើអ្នកធ្វើការងារដែលមានសកម្មភាពខ្លាំងក្លាបែបនេះប៉ុន្មានថ្ងៃក្នុងមួយអាទិត្យ? | ចំនួនថ្ងៃ └─┴─┘ |
| Q 38 | តើអ្នកចំណាយរយៈពេលប៉ុន្មានក្នុងមួយថ្ងៃ ដើម្បីធ្វើការងារដែលមានសកម្មភាពខ្លាំងក្លា បែបនេះ? | ម៉ោង:នាទី └─┴─┘:└─┴─┘ |
| Q 39 | តើការងារដែលអ្នកធ្វើមានសកម្ម​ភាពមធ្យមអាចធ្វើឲ្យអ្នកហត់ ឬបេះដូងដើរញាប់ល្មម ដូចជា ដើរយ៉ាងលឿន ដាំ​ឬប្រមូលដំណាំកសិផល  បោសសម្អាត បោកគក់សំលៀកបំពាក់ដោយដៃ ឬជិះកង់ យ៉ាងតិច ១០នាទី ជាប់ៗគ្នាដែរឬទេ?  *(សូមបង្ហាញផ្ទាំងរូបភាព)* | 1. បាទ/ចាស 2. ទេ (រំលងទៅ Q 41) |
| Q 40 | តើអ្នកធ្វើការងារដែលមានសកម្មភាពមធ្យមបែបនេះប៉ុន្មានថ្ងៃក្នុងមួយអាទិត្យ? | ចំនួនថ្ងៃ └─┴─┘ |
| Q 41 | តើអ្នកចំណាយរយៈពេលប៉ុន្មានក្នុងមួយថ្ងៃដើម្បីធ្វើការងារដែលមានសកម្មភាពមធ្យមបែបនេះ? | ម៉ោង:នាទី └─┴─┘:└─┴─┘ |

| ការធ្វើដំណើរ | | |
| --- | --- | --- |
| នៅក្នុងសំណួរខាងក្រោមនេះមិនរាប់បញ្ចូលកាយវប្បកម្ម នៅពេលប្រកបការងារដែលបានរៀបរាប់រួចមកហើយទេ។ ពេលនេះ យើងខ្ញុំចង់សួរអ្នកអំពីមធ្យោបាយធ្វើដំណើរដែលអ្នកតែងតែប្រើប្រាស់ជាធម្មតាពីកន្លែងមួយទៅកន្លែងមួយ ដូចជាទៅធ្វើការ ទៅផ្សារទិញឥវ៉ាន់ ទៅវត្តអារាម/វិហារជាដើម។ | | |
| កូដ | សំណួរ | ចម្លើយ |
| Q 42 | តើអ្នកដើរ ឬជិះកង់ពីកន្លែងមួយទៅកន្លែងមួយ យ៉ាងតិចណាស់ ១០នាទី ជាប់ៗគ្នាដែរឬទេ? | 1. បាទ/ចាស 2. ទេ (រំលងទៅ Q 45) |
| Q 43 | តើមានប៉ុន្មានថ្ងៃក្នុងមួយអាទិត្យដែលអ្នកធ្វើដំណើរពីកន្លែងមួយទៅកន្លែងមួយ ដោយថ្មើរជើង ឬជិះកង់ យ៉ាងតិចណាស់១០នាទីជាប់ៗគ្នា? | ចំនួនថ្ងៃ └─┴─┘ |
| Q 44 | តើអ្នកចំណាយពេលប៉ុន្មាន សម្រាប់ការធ្វើដំណើរដោយ ដើរ ឬជិះកង់ក្នុងមួយថ្ងៃ? | ម៉ោង:នាទី └─┴─┘:└─┴─┘ |

| **សកម្មភាពកំសាន្ត** | | |
| --- | --- | --- |
| សំណួរខាងក្រោមនេះ មិនរាប់បញ្ចូលសកម្មភាពដែលធ្វើការ និងពេលធ្វើដំណើរទេ ។ ពេលនេះ យើងខ្ញុំចង់សួរអ្នកអំពីកីឡា ការហាត់ប្រាណដើម្បីសម្រករាង និងសកម្មភាពកំសាន្តផ្សេងៗ។ | | |
| កូដ | សំណួរ | ចម្លើយ |
| Q 45 | តើអ្នកបានលេងកីឡា ហាត់ប្រាណ ឬសកម្មភាពកំសាន្ត ដែលមានសកម្មភាពខ្លាំងក្លាធ្វើឲ្យចង្វាក់ដង្ហើម ឬបេះដូងដើរញាប់ខ្លាំងជាងធម្មតា ដូចជាការរត់ ការលេងបាល់ទាត់ យ៉ាងតិចណាស់ ១០នាទីជាប់ៗគ្នាដែរឬទេ?  *(សូមបង្ហាញផ្ទាំងរូបភាព)* | 1. បាទ/ចាស 2. ទេ (Go to Q 48) |
| Q 46 | តើអ្នកបានលេងកីឡា ហាត់ប្រាណ ឬសកម្មភាពកំសាន្ត ដែលមានសកម្មភាពខ្លាំងក្លាបែបនេះប៉ុន្មានថ្ងៃក្នុងមួយអាទិត្យ? | ចំនួនថ្ងៃ └─┴─┘ |
| Q 47 | តើអ្នកចំណាយរយៈពេលប៉ុន្មានក្នុងមួយថ្ងៃសំរាប់លេងកីឡា ហាត់ប្រាណ ឬសកម្មភាពកំសាន្ត​ដែលមានសកម្មភាព  ខ្លាំងក្លារបៀបនេះ? | ម៉ោង:នាទី └─┴─┘:└─┴─┘ |
| Q 48 | តើអ្នកបានលេងកីឡា ហាត់ប្រាណ ឬសកម្មភាពកំសាន្ត ដែលមានសកម្មភាពល្មម ធ្វើឲ្យចង្វាក់ដង្ហើម ឬបេះដូងដើរញាប់ខ្លាំងជាងធម្មតាបន្តិចបន្តួច ដូចជា ជិះកង់ ហែលទឹក ឬលេងបាល់បោះ យ៉ាងតិចណាស់ ១០នាទី ជាប់ៗគ្នាដែរឬទេ? | 1. បាទ/ចាស 2. ទេ (រំលងទៅ Q 51) |
| Q 49 | តើអ្នកបានលេងកីឡា ហាត់ប្រាណ ឬសកម្មភាពកំសាន្ត ដែលមានសកម្មភាពល្មមបែបនេះប៉ុន្មានថ្ងៃក្នុងមួយអាទិត្យ? | ចំនួនថ្ងៃ └─┴─┘ |
| Q 50 | តើអ្នកចំណាយរយៈពេលប៉ុន្មានក្នុងមួយថ្ងៃសំរាប់លេងកីឡា ហាត់ប្រាណ ឬសកម្មភាពកំសាន្តដែលមានសកម្មភាពល្មមបែបនេះ? | ម៉ោង:នាទី └─┴─┘:└─┴─┘ |

| ឥរិយាបថអង្គុយយូរនៅមួយកន្លែង | | |
| --- | --- | --- |
| យើងនឹងសាកសួរអ្នកនូវសំណួរខាងក្រោមអំពីការអង្គុយឬទម្រេតខ្លួននៅក្នុងការិយាល័យធ្វើការ នៅផ្ទះ ការធ្វើដំណើរពីកន្លែងមួយទៅកន្លែងមួយ ឬជាមួយមិត្តភ័ក្តិ រួមបញ្ចូលទាំងការអង្គុយនៅតុការិយាល័យ អង្គុយជាមួយមិត្តភ័ក្តិ ធ្វើដំណើរដោយម៉ូតូ ទុគៗ នៅក្នុងឡានផ្ទាល់ខ្លួន ឡានក្រុង រថភ្លើង អង្គុយអានសៀវភៅ អង្គុយលេងបៀរ ឬមើលទូរទស្សន៍ ប៉ុន្តែមិនរាប់បញ្ចូលពេលវេលាដែលអ្នកគេងឡើយ ។ *(សូមបង្ហាញផ្ទាំងរូបភាព)* | | |
| កូដ | សំណួរ | ចម្លើយ |
| Q 51 | តើអ្នកចំណាយពេលប៉ុន្មានក្នុងការ អង្គុយស្ងៀម ឬទម្រេតខ្លួន ក្នុងមួយថ្ងៃ ? | ម៉ោង:នាទី └─┴─┘:└─┴─┘ |

*៥) កម្រងសំណួរមបន្ថែម*

| ប្រវត្តិនៃការឡើងសម្ពាធឈាម | | |
| --- | --- | --- |
| កូដ | សំណួរ | ចម្លើយ |
| Q 52 | តើអ្នកធ្លាប់ឲ្យវេជ្ជបណ្ឌិត ឬបុគ្គលិកសុខាភិបាលវាស់សម្ពាធឈាមអ្នកដែរឬទេ? | 1. បាទ/ចាស 2. ទេ (រំលងទៅ Q 57) |
| Q 53 | តើអ្នកត្រូវបានវេជ្ជបណ្ឌិត ឬបុគ្គលិកសុខាភិបាលប្រាប់ពីការលើសម្ពាធឈាម នៅក្នុងពេល១២ខែ កន្លងមកនេះដែរឬទេ? | 1. បាទ/ចាស 2. ទេ (រំលងទៅ Q 57) |
| Q 54 | ក្នុងរយៈពេល ២អាទិត្យ កន្លងមកនេះ តើអ្នកបានលេបថ្នាំសម្រាប់បញ្ចុះសម្ពាធឈាម ដែលចេញវេជ្ជបញ្ជាដោយវេជ្ជបណ្ឌិត ឬបុគ្គលិក  សុខាភិបាលដែរឬទេ? | 1. បាទ/ចាស 2. ទេ |
| Q 55 | តើអ្នកធ្លាប់ទៅជួបជាមួយគ្រូខ្មែរដើម្បីព្យាបាលការឡើងសម្ពាធឈាម ឬជំងឺលើសសម្ពាធឈាមដែរឬទេ? | 1. បាទ/ចាស 2. ទេ |
| Q 56 | តើអ្នកកំពុងប្រើថ្នាំខ្មែរដើម្បីព្យាបាលការឡើងសម្ពាធឈាមដែរឬទេ? | 1. បាទ/ចាស 2. ទេ |

| ប្រវត្តិជំងឺទឹកនោមផ្អែម | | |
| --- | --- | --- |
| កូដ | សំណួរ | ចម្លើយ |
| Q 57 | តើអ្នកធ្លាប់ឲ្យវេជ្ជបណ្ឌិត ឬបុគ្គលិកសុខាភិបាលពិនិត្យឈាមមើលកំរិតជាតិស្ករដែរឬទេ? | 1. បាទ/ចាស 2. ទេ (រំលងទៅ Q 63) |
| Q 58 | តើវេជ្ជបណ្ឌិត ឬបុគ្គលិកសុខាភិបាលធ្លាប់បានប្រាប់អ្នកថាអ្នកឡើងជាតិស្ករនៅក្នុងឈាម ឬមានជំងឺទឹកនោមផ្អែមដែរឬទេ? | 1. បាទ/ចាស 2. ទេ |
| Q 59 | តើវេជ្ជបណ្ឌិត ឬបុគ្គលិកសុខាភិបាលបានប្រាប់ថាអ្នកឡើងជាតិស្ករនៅក្នុងឈាម ឬមានជំងឺទឹកនោមផ្អែម ក្នុងរយៈពេល ១២ ខែកន្លងមកនេះដែរឬទេ? | 1. បាទ/ចាស 2. ទេ |
| Q 60 | ក្នុងរយៈពេល ២អាទិត្យ កន្លងមកនេះ តើអ្នកបានលេបថ្នាំសម្រាប់ជំងឺទឹកនោមផ្អែម ដែលចេញវេជ្ជបញ្ជាដោយវេជ្ជបណ្ឌិត ឬបុគ្គលិកសុខាភិបាល ដែរឬទេ? | 1. បាទ/ចាស 2. ទេ |
| Q 61 | តើអ្នកកំពុងចាក់ថ្នាំអាំងស៊ុយលីនសម្រាប់ជំងឺទឹកនោមផ្អែម ដែលចេញវេជ្ជបញ្ជាដោយវេជ្ជបណ្ឌិត ឬបុគ្គលិកសុខាភិបាល ដែរឬទេ? | 1. បាទ/ចាស 2. ទេ |
| Q 62 | តើអ្នកកំពុងប្រើថ្នាំខ្មែរដើម្បីព្យាបាលជំងឺទឹកនោមផ្អែម ដែរឬទេ? | 1. បាទ/ចាស 2. ទេ |

| ***ប្រវត្តិនៃការឡើងជាតិខ្លាញ់កូឡេស្តេរ៉ូលនៅក្នុងឈាម*** | | |
| --- | --- | --- |
| កូដ | សំណួរ | ចម្លើយ |
| Q 63 | តើអ្នកធ្លាប់ឲ្យវេជ្ជបណ្ឌិត ឬបុគ្គលិកសុខាភិបាលពិនិត្យឈាមមើលកំរិតជាតិខ្លាញ់កូឡេស្តេរ៉ូលរបស់អ្នក (ជាតិខ្លាញ់នៅក្នុងឈាម) ដែរឬទេ? | 1. បាទ/ចាស 2. ទេ (រំលងទៅ Q 68) |
| Q 64 | តើវេជ្ជបណ្ឌិត ឬបុគ្គលិកសុខាភិបាលធ្លាប់បានប្រាប់អ្នកថាអ្នកមានជាតិខ្លាញ់នៅក្នុងឈាមកើនឡើងដែរឬទេ? | 1. បាទ/ចាស 2. ទេ (រំលងទៅ Q 68) |
| Q 65 | តើវេជ្ជបណ្ឌិត ឬបុគ្គលិកសុខាភិបាលបានប្រាប់អ្នកថាមានជាតិខ្លាញ់នៅក្នុងឈាមកើនឡើង ក្នុងរយៈពេល ១២ខែ កន្លងមកនេះ ដែរឬទេ? | 1. បាទ/ចាស 2. ទេ |
| Q 66 | ក្នុងរយៈពេល ២អាទិត្យ កន្លងមកនេះ តើអ្នកបានលេបថ្នាំសម្រាប់ការឡើងជាតិខ្លាញ់នៅក្នុងឈាម ដែលចេញវេជ្ជបញ្ជាដោយវេជ្ជបណ្ឌិត ឬបុគ្គលិកសុខាភិបាល ដែរឬទេ? | 1. បាទ/ចាស 2. ទេ |
| Q 67 | តើអ្នកកំពុងប្រើថ្នាំខ្មែរដើម្បីព្យាបាលការឡើងជាតិខ្លាញ់នៅក្នុងឈាមដែរឬទេ? | 1. បាទ/ចាស 2. ទេ |

| ***ការផ្តល់ដំបូន្មានអំពីរបៀបរបបរស់នៅ*** | | |
| --- | --- | --- |
| Q 68: ក្នុងរយៈពេល៣ឆ្នាំកន្លងមកនេះ តើមានវេជ្ជបណ្ឌិត​ ឬបុគ្គលិកសុខាភិបាលណាម្នាក់បានផ្តល់ដំបូន្មានអ្នក​ឲ្យធ្វើនូវចំណុចណាមួយដូចខាងក្រោមនេះដែរឬទេ? (សូមកត់ត្រាចម្លើយ នីមួយៗ) | | |
| កូដ | សំណួរ | ចម្លើយ |
| Q 68.1 | ឈប់ជក់បារី ឬមិនឲ្យចាប់ផ្តើមជក់បារី | 1. បាទ/ចាស 2. ទេ |
| Q 68.2 | កាត់បន្ថយបរិមាណជាតិអំបិលនៅក្នុងម្ហូបអាហារ | 1. បាទ/ចាស 2. ទេ |
| Q 68.3 | ទទួលទានបន្លែ ឬផ្លែឈើយ៉ាងហោចឲ្យបាន ៥រង្វាល់ក្នុងមួយថ្ងៃ | 1. បាទ/ចាស 2. ទេ |
| Q 68.4 | កាត់បន្ថយជាតិខ្លាញ់នៅក្នុងរបបអាហារ | 1. បាទ/ចាស 2. ទេ |
| Q 68.5 | ចាប់ផ្តើម ឬធ្វើកាយវប្បកម្មឲ្យបានច្រើន | 1. បាទ/ចាស 2. ទេ |
| Q 68.6 | រក្សាទម្ងន់ខ្លួនឲ្យមានសុខភាពល្អ កុំឲ្យលើសទម្ងន់ និងធាត់ ឬឲ្យសម្រកទម្ងន់ | 1. បាទ/ចាស 2. ទេ |

| ប្រវត្តិគ្រួសារដែលទាក់ទងទៅនឹងជំងឺមិនឆ្លង និងការលេបថ្នាំពន្យារកំណើត | | |
| --- | --- | --- |
| កូដ | សំណួរ | ចម្លើយ |
| Q 69 | តើអ្នកធ្លាប់ដឹងដែរឬទេថាមានអ្នកណាម្នាក់នៅក្នុងគ្រួសាររបស់អ្នកមានការលើសជាតិស្ករក្នុងឈាម ឬទឹកនោមផែ្អម? | 1. បាទ/ចាស 2. ទេ   77. មិនដឹង |
| Q 70 | តើអ្នកធ្លាប់ដឹងដែរឬទេថាមានអ្នកណាម្នាក់នៅក្នុងគ្រួសាររបស់អ្នកមាន*ការឡើងជាតិខ្លាញ់កូឡេស្តេរ៉ូល នៅក្នុងឈាម*? | 1. បាទ/ចាស 2. ទេ   77. មិនដឹង |
| Q 71 | តើអ្នកធ្លាប់ដឹងដែរឬទេថាមានអ្នកណាម្នាក់នៅក្នុងគ្រួសាររបស់អ្នកមាន*ការ*ការលើសម្ពាធឈាម? | 1. បាទ/ចាស 2. ទេ   77. មិនដឹង |
| Q 72 | តើអ្នកធ្លាប់ដឹងដែរឬទេថាមានអ្នកណាម្នាក់នៅក្នុងគ្រួសាររបស់អ្នកធ្លាប់គាំងបេះដូង ឬចុកដើមទ្រូងដោយសារជំងឺបេះដូង ឬដាច់សរសៃឈាមក្នុងខួរក្បាល ដែរឬទេ? | 1. បាទ/ចាស 2. ទេ   77. មិនដឹង |
| Q 73 | តើអ្នកកំពុងលេបថ្នាំពន្យារកំណើតគ្រាប់ដែរឬទេ?  **(សួរចំពោះតែស្រ្តីប៉ុណ្ណោះ)** | 1. ចាស 2. ទេ |
| Q 74 | តើអ្នកដឹងដែរឬទេថាអ្នកពេលកើត មុនខែ ឬគ្រប់ខែ? | 1. កើតមិនគ្រប់ខែ  2. កើតគ្រប់ខែ  77. មិនដឹង |

ស្តេប ២**:** ការវាស់សម្ពាធឈាម កម្ពស់ និងថ្លឹងទម្ងន់

| **លេខកូដសម្គាល់ខ្លួនរបស់**អ្នកសម្ភាស | | └─┴─┘ |
| --- | --- | --- |
| **Height, Weight and Waist and Hip Circumference** | | |
| កូដ | សំណួរ | ចម្លើយ |
| Q 75 | លេខកូដសម្គាល់ឧបករណ៍វាស់កម្ពស់ និងថ្លឹងទម្ងន់ | ម៉ែត្រសំពត់ └─┴─┘  ជញ្ជីង └─┴─┘ |
| Q 76 | កម្ពស់ | └─┴─┴ . ┴─┘ស.ម |
| Q 77 | ទម្ងន់ | └─┴─┴ . ┴─┘គ.ក្រ |
| Q 78 | លេខកូដសម្គាល់ឧបករណ៍វាស់ទំហំចង្កេះ | └─┴─┘ |
| Q 79 | ទំហំចង្កេះ | └─┴─┴ . ┴─┘ស.ម |
| Q 80 | ទំហំត្រគាក | └─┴─┴ . ┴─┘ស.ម |
| ការវាស់សម្ពាធឈាម (វាស់ពីលើកទីមួយទៅមួយលើកទៀតត្រូវរងចាំប្រហែល៥នាទី) | | |
| Q 81 | លេខកូដសម្គាល់ម៉ាស៊ីនវាស់សម្ពាធឈាម | └─┴─┘ |
| Q 82 | វាស់លើកទី ១ | ស៊ីស្តូលីក └─┴─┴─┘ mmHg  ដ្យាស្តូលីក └─┴─┴─┘ mmHg |
| Q 83 | វាស់លើកទី ២ | ស៊ីស្តូលីក └─┴─┴─┘ mmHg  ដ្យាស្តូលីក └─┴─┴─┘ mmHg |
| Q 84 | វាស់លើកទី ៣ | ស៊ីស្តូលីក └─┴─┴─┘ mmHg  ដ្យាស្តូលីក └─┴─┴─┘ mmHg |
| Q 85 | ក្នុងរយៈពេល ២អាទិត្យ កន្លងមកនេះ តើអ្នកបានលេបថ្នាំព្យាបាលការឡើងសម្ពាធឈាម ដែលចេញវេជ្ជបញ្ជាដោយវេជ្ជបណ្ឌិត ឬបុគ្គលិកសុខាភិបាលដែរឬទេ? | 1. បាទ/ចាស 2. ទេ |

ស្តេប ៣**:** ការជោះឈាមដើម្បីពិនិត្យជាតិស្ករ និងជាតិខ្លាញ់

| **លេខកូដសម្គាល់ខ្លួនរបស់អ្នកសម្ភាស** | | └─┴─┘ |
| --- | --- | --- |
| **Code** | **Question** | **Response** |
| កម្រិតជាតិស្ករនៅក្នុងឈាម | | |
| Q 86 | ក្នុងរយៈពេល ១២ម៉ោង កន្លងមកនេះ តើអ្នកបានទទួលទាន ឬផឹកអ្វីក្រៅពីទឹកដែរឬទេ? | 1. បាទ/ចាស 2. ទេ |
| Q 87 | លេខកូដសម្គាល់ម៉ាស៊ីនវាស់កម្រិតជាតិស្ករនៅក្នុងឈាម | └─┴─┘ |
| Q 88 | ម៉ោងជោះឈាម (គិត 24 ម៉ោងក្នុងមួយថ្ងៃ) | ម៉ោង:នាទី └─┴─┘:└─┴─┘ |
| Q 89 | កម្រិតជាតិស្ករនៅក្នុងឈាមពេលមិនទាន់បានបរិភោគឬផឹកអ្វី ក្រៅពីទឹក | └─┴─┴. └─┴─┴ mmol/l ឬ mg/dl |
| កម្រិតជាតិខ្លាញ់នៅក្នុងឈាម | | |
| Q 90 | លេខកូដសម្គាល់ម៉ាស៊ីន | └─┴─┘ |
| Q 91 | កម្រិតជាតិខ្លាញ់នៅក្នុងឈាមសរុប | └─┴─┴. └─┴─┴ mmol/l ឬ mg/dl |
| **បញ្ចប់ចំពោះអ្នកចូលរួមដែលមកធ្វើតេស្តឈាមរកមេរោគអេដស៍**  ** **សូមអរគុណដែលបានចូលរួមក្នុងការអង្កេតមួយនេះ** ** | | |

ផ្នែកទី២៖ អ្នករស់នៅជាមួយមេរោគអេដស៍ និងថ្នាំពន្យារការវិវឌ្ឍន៏របស់ជំងឺ

| **សួរសំណួរខាងក្រោមចំពោះតែអ្នកចូលរួមដែលមកបើកថ្នាំពន្យារជំងឺអេដស៍** | | |
| --- | --- | --- |
| **លេខកូដសម្គាល់ខ្លួនរបស់អ្នកសម្ភាស** | | └─┴─┘ |
| **Code** | **Questions** | **Response** |
| Q 92 | តើអ្នករស់នៅជាមួយជំងឺអេដស៍បានប៉ុន្មានខែ ឬឆ្នាំហើយ?  *(កត់ត្រាចម្លើយតែមួយគត់ មិនមែនកត់ត្រាទាំងពីរនោះទេ)* | ចំនួនខែ└─┴─┘  ចំនួនឆ្នាំ└─┴─┘ |
| Q 93 | តើអ្នកបានប្រើប្រាស់ថ្នាំពន្យារជំងឺអេដស៍បាន ប៉ុន្មានខែ ឬឆ្នាំហើយ?  *(កត់ត្រាចម្លើយតែមួយគត់ មិនមែនកត់ត្រាទាំងពីរនោះទេ)* | ចំនួនខែ└─┴─┘  ចំនួនឆ្នាំ└─┴─┘ |
| Q 94 | ប្រភេទថ្នាំពន្យារជំងឺអេដស៍កំពុងលេបបច្ចុប្បន្ន  *(សូមពិនិត្យមើលក្នុងកូនសៀវភៅតាមដានជំងឺរបស់គាត់)* | ប្រភេទថ្នាំដែលគាត់កំពុងលេបរាល់ថ្ងៃ  …………………………………….  ថ្ងៃ ខែ ឆ្នាំចាប់ផ្តើម  └─┴─┘└─┴─┘└─┴─┴─┘ |
| Q 95 | ប្រភេទថ្នាំពន្យារជំងឺអេដស៍ធ្លាប់លេបពីមុន  *(សូមពិនិត្យមើលក្នុងកូនសៀវភៅតាមដានជំងឺរបស់គាត់)* | ប្រភេទថ្នាំដែលគាត់លេបពីមុន  …………………………………….  ថ្ងៃ ខែ ឆ្នាំចាប់ផ្តើម  └─┴─┘└─┴─┘└─┴─┴─┘ |
| Q 96 | តើអ្នកកំពុងតែប្រើប្រាស់ថ្នាំដើម្បីការពារ ឬព្យាបាលជំងឺឱកាសនិយមផ្សេងៗដែរឬទេ? | 1. បាទ/ចាស 2. ទេ   77. មិនដឹង |
| Q 97 | តើអ្នកកំពុងតែប្រើប្រាស់ថ្នាំដើម្បីព្យាបាលជំងឺរបេងដែរឬទេ? | 1. បាទ/ចាស 2. ទេ |
| Q 98 | តើក្នុងរយៈពេល៦ខែចុងក្រោយនេះ អ្នកបាន  ពិនិត្រឈាមរាប់មើលចំនួនកោសិកាគ្រាប់ឈាមស CD4 ដែរឬទេ? | 1. បាទ/ចាស 2. ទេ (បើ ទេ សូមបញ្ចប់ការសម្ភាស) |
| Q 99 | តើនៅក្នុងលទ្ធផលពិនិត្រឈាមចុងក្រាយបំផុតចំនួនកោសិកាគ្រាប់ឈាមស CD4 របស់អ្នកមានចំនួនប៉ុន្មានដែរ? | ចំនួន└─┴─┴─┘  (00 បើមិនដឹង) |
| ** **សូមអរគុណដែលបានចូលរួមក្នុងការអង្កេតមួយនេះ** ** | | |
